# Toward an idiographic understanding of the role of sleep‐mood dynamics in adolescents' internalizing symptoms

**DOI:** 10.1002/jcv2.70082

**Published:** 2025-12-17

**Authors:** Konstantin Drexl, Setareh Ranjbar, Sébastien Urben, Kerstin Jessica Plessen, Jennifer Glaus

**Affiliations:** ^1^ Division for Child and Adolescent Psychiatry Department of Psychiatry Lausanne University Hospital and University of Lausanne Lausanne Switzerland; ^2^ Faculty of Psychology and Educational Sciences Clinical Psychology Unit for Intellectual and Developmental Disabilities University of Geneva Geneva Switzerland; ^3^ Department of Psychiatry Center for Psychiatric Epidemiology and Psychopathology Lausanne University Hospital and University of Lausanne Prilly Switzerland

**Keywords:** actigraphy, adolescence, ecological momentary assessment, sleep

## Abstract

**Background:**

Adolescence is marked by increased vulnerability to sleep disturbances and mood disorders. Understanding how day‐to‐day changes in sleep and mood are linked within the same individual is crucial for clarifying sleep's role in emerging internalizing disorders. However, the extent to which an adolescent's fluctuations in sleep predict next‐day mood may differ across individuals. This study examined such differences in person‐specific sleep‐mood dynamics and their links with depression and anxiety symptoms.

**Methods:**

A total of 113 Swiss adolescents (57% female, mean age = 15.4 years, SD = 0.97) participated in three 3‐week waves of combined actigraphy, four daily Ecological Momentary Assessments (EMA), and weekly reports of depressive and anxiety symptoms. Bayesian multilevel models estimated person‐specific associations across two mood dimensions (sad‐vs‐happy and anxious‐vs‐calm) combined with sleep duration (SDur), sleep midpoint (SMid), and sleep regularity (SReg; i.e., overlap between each daily cycle and the participant's most typical cycle). Bayesian hypothesis tests examined evidence for differences in person‐specific associations and whether these differences predicted internalizing symptoms.

**Results:**

Person‐specific associations involving SDur or SMid showed within‐person effects at the group‐level, but no convincing evidence for individual differences in this dynamic. In contrast, there were clear individual differences in how SReg related to anxious‐vs‐calm mood (*τ* ^2^ = 0.47, [0.09; 0.79], BF_10_ = 13.2). Adolescents whose calm mood was more strongly tied to a regular sleep–wake cycle reported fewer anxiety symptoms (*b* = 3.57, 95%CrI [‐3.67; 9.31], BF_01_ = 7.69).

**Conclusion:**

Our findings suggest that adolescents whose daytime mood is closely coupled with regular sleep–wake patterns may experience protection against anxiety symptoms. They also show that adolescents differ meaningfully in these sleep–mood associations, challenging conventional group‐level approaches. Future work should extend this idiographic approach to more diverse populations and longer timescales to clarify the role of sleep–mood dynamics in internalizing disorders.

## Key Points

**What's known?**

- Adolescence is a period of elevated risk for both internalizing symptoms and sleep disturbances, suggesting a dynamic role of sleep as a modifiable risk factor for emerging internalizing disorders.

**What's new?**

- This study highlights significant differences in how regularity of the sleep‐wake cycle predicts next‐day mood among adolescents, challenging prior generalizations of the sleep‐mood interplay.
- Differences in person‐specific associations between sleep regularity (SReg) and calm mood predicted symptoms of anxiety.

**What's relevant?**

- Co‐fluctuation of mood and SReg may emerge as a dynamic marker for risk‐and‐resilience processes.
- Further research is necessary to explore the heterogeneity in diverse populations and inform effective preventive strategies for adolescent mood disorders.

## Graphical abstract

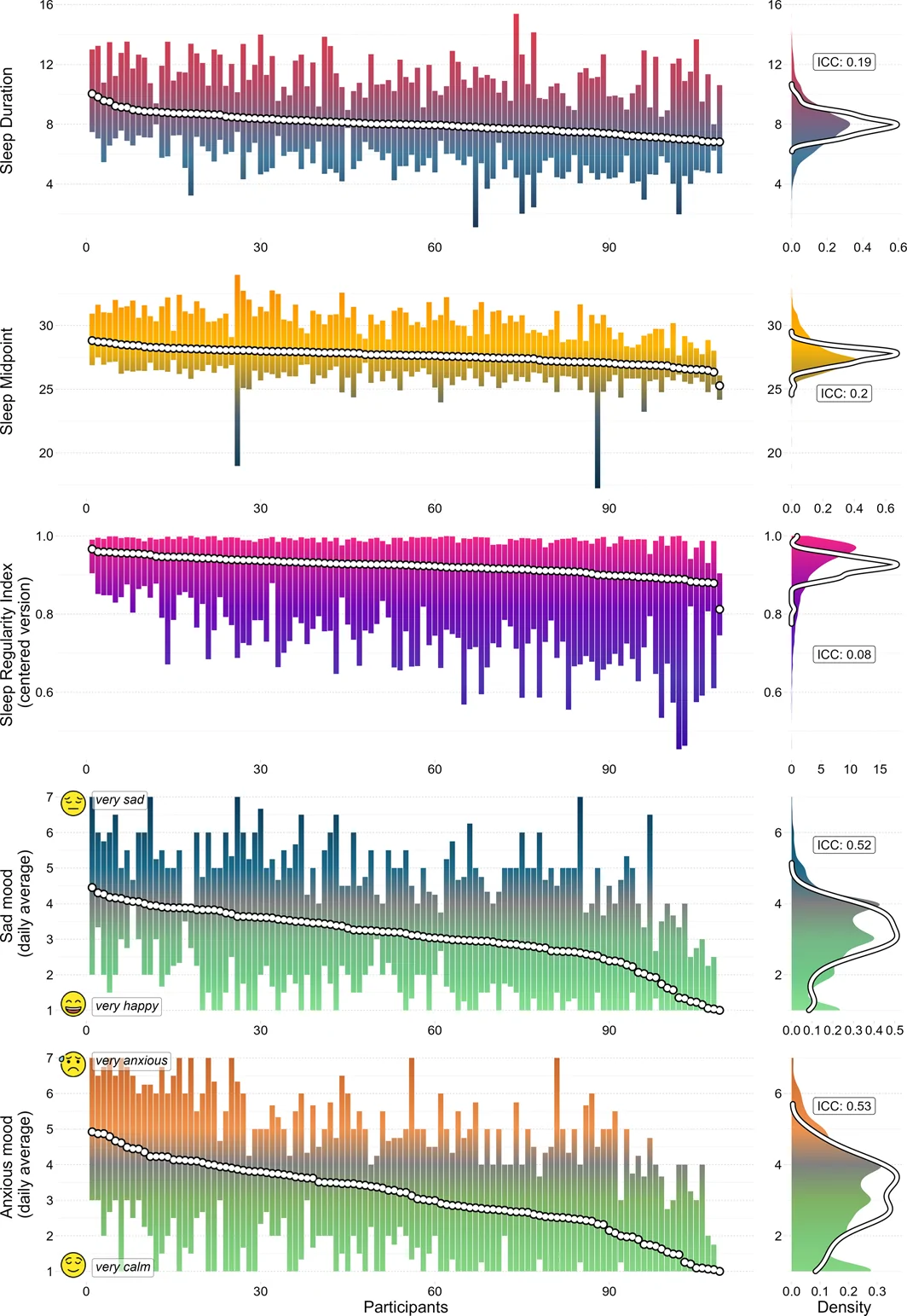

## INTRODUCTION

Adolescence is a critical developmental period marked by heightened emotional sensitivity and an increased risk for internalizing disorders, such as depression and anxiety (Casey et al., 2008). Together, these disorders contribute significantly to the global psychiatric burden, often leading to long‐term consequences such as years lived with disability (GBD, 2019 Mental Disorders Collaborators, 2022), reduced personal fulfillment (Essau et al., 2014), and lower educational attainment (Clayborne et al., 2019), particularly if left untreated (Rice et al., 2017). Consequently, adolescence represents a pivotal window for preventive interventions targeting modifiable factors of risk and resilience such as sleep (Fusar‐Poli et al., 2021; Krokstad et al., 2024). To advance a granular understanding of the role of sleep in emerging internalizing disorders, research should increasingly consider heterogeneity in etiological mechanisms contributing to developmental trajectories (Vidal‐Ribas, 2025). Investigating idiographic (i.e., person‐specific) dynamics of the sleep‐mood relationship may therefore provide valuable insights into the emergence of internalizing disorders in adolescents.

Adolescence is a peak period for mental‐health challenges, coinciding with significant changes in sleep physiology. Neurobiological changes delay the onset of melatonin secretion (Crowley et al., 2018) and slow the accumulation of sleep pressure (Gradisar et al., 2022; Skorucak et al., 2021), increasing evening alertness and making it progressively more difficult to fall asleep early (Monterastelli et al., 2024; Skeldon et al., 2016). This delay in the biological clock often conflicts with early school start times, reducing sleep duration (SDur) and leading to accumulated sleep debt over the school week (Gariepy et al., 2020; Putilov, 2023). Such chronic discrepancies create significant social jetlag, defined as misalignment between biological rhythms and societal schedules (Roenneberg et al., 2019). These combined bioregulatory, psychosocial, and societal pressures have been aptly described as a “perfect storm” for disturbed circadian rhythms ultimately leading to adverse health outcomes, particularly in mental health (Crowley et al., 2018).

Individual differences in circadian timing appear to influence the susceptibility to psychiatric disorders. Adolescents reporting greater social jetlag (Mathew et al., 2019; Tamura & Okamura, 2024) or a stronger preference for later bedtimes (i.e., late chronotypes) are more likely to experience symptoms of depression and anxiety (Bei et al., 2017; Brown et al., 2018; Lok et al., 2024; Messman et al., 2024). Irregular circadian rhythms and sleep disturbances also characterize emerging mood disorders (Castiglione‐Fontanellaz et al., 2023; Kelly et al., 2022; Lunsford‐Avery et al., 2022). Psychological factors such as insomnia, rumination, and pre‐sleep arousal may create vicious cycles of worsening sleep and mood (Alfano et al., 2010; Gradisar et al., 2022; Maskevich et al., 2021; Riemann et al., 2024). These mechanisms interact with physiological, developmental changes (Galván, 2020), as well as environmental and social stressors, amplifying risk for internalizing disorders among adolescents (Blake, Trinder, & Allen, 2018; Crouse et al., 2021).

Multiple research lines emphasize the critical role of sleep disturbances in shaping affective functioning and internalizing symptom trajectories. Meta‐analyses of prospective studies indicate that sleep disturbances commonly precede depressive symptoms (Lovato & Gradisar, 2014). Moreover, meta‐analyses on experimental research confirm that sleep restriction and deprivation negatively affect daytime mood, emotional reactions, and emotion regulation in adolescents (Palmer et al., 2024; Tomaso et al., 2021). However, these experimentally induced sleep restrictions may differ markedly from the natural fluctuations in adolescents' sleep behavior. Consequently, it remains unclear whether equivocal effects apply to every adolescent in fully naturalistic settings, where the individual must balance biological, social, and academic demands. One study provided a seminal illustration of contradictory nature of these factors revealing that internalizing symptoms were minimal in adolescents who slept on average 8.75 h, whereas academic performance peaked in adolescents sleeping between 7 and 7.5 h (Fuligni et al., 2018).

However, instead of comparing sleep patterns and affective functioning across individuals, a process‐focused developmental approach is interested in how intraindividual sleep–mood dynamics unfold over various timescales within the same individual to influence clinical trajectories in affective functioning and internalizing symptoms (Becker et al., 2017). The most frequently investigated timescale of the sleep‐mood relationship is probably the daily level, recently synthesized in a systematic review encompassing 121 studies, including 26 adolescent samples (Hickman et al., 2024). Unsurprisingly, the review highlighted substantial inconsistencies with contradictory findings interpreted as the result of heterogeneous monitoring methods (e.g., actigraphy, daily diaries, and Ecological Momentary Assessment [EMA]), as well as inconsistent operationalization of sleep and mood variables. Nonetheless, the reviewers concluded that changes in sleep patterns predicted next‐day mood more robustly than vice versa, with SDur demonstrating the strongest associations (Hickman et al., 2024).

Despite growing interest in naturalistic within‐person findings, individual differences in adolescents' sleep‐mood associations remain largely unaddressed, partly due to methodological challenges (e.g., convergence issues when estimating the variation in random slopes; Chachos et al., 2023; Könen et al., 2016; Neubauer et al., 2020; Shen et al., 2022). Nonetheless, adolescents may differ markedly in their daily sleep‐mood covariation, suggesting some individuals may be more sensitive to sleep disruptions than others. Multiple generic terms have been used to describe these individual within‐person associations, including “person‐specific associations” (Hallensleben et al., 2024), “idiographic associations” (Kraiss et al., 2024), “reactivity” (Messman et al., 2023), and “random slopes” (Elmer et al., 2023), or “within‐person couplings (WPC)” (Neubauer & Schmiedek, 2020). The latter reflects the idea that sleep and mood can be coupled at different intensities, directions, and across multiple timescales. Of note, we consider these associations as the temporal signatures rather than direct indicators of the underlying and by far more complex, mechanisms (Blake, Trinder, & Allen, 2018; Haslbeck & Ryan, 2022). However, for the present paper we decided to adopt the term “person‐specific associations”, which is more accessible for the wider research community. More specifically, we focus person‐specific sleep‐mood associations on the initial timescale of interest, that is, on the daily level, though higher‐level fluctuations may be equally informative for internalizing etiology (Vidal Bustamante et al., 2020).

Understanding idiographic sleep–mood dynamics may help identify adolescents vulnerable to negative reinforcement cycles, where poor sleep worsens daytime affective functioning and escalates into emotional disorders characterized by persistent sleep disturbances (Alvaro et al., 2013; Blake, Trinder, & Allen, 2018; Lovato & Gradisar, 2014; Nutt et al., 2008). Identifying these dynamic markers linked to internalizing symptom development could also facilitate targeted sleep‐based interventions for adolescents most likely to benefit quickly and effectively.

### Current study

This study is part of the mSanté project, an intensive longitudinal study with adolescents tracking sleep and daytime mood over an extended period (Drexl et al., 2024). Our first objective was to quantify differences among person‐specific associations of sleep patterns and next‐day mood by systematically combining two mood dimensions, sad and anxious mood, with three sleep patterns: SDur (i.e., receiving more vs. less sleep than usual), timing (i.e., placing the midpoint of the major sleep period earlier vs. later than usual), and the regularity of the sleep‐wake cycle (i.e., the overlap of a given 24 h cycle with the most typical cycle). We registered the following hypothesis (Drexl et al., 2023):

H 1 Adolescents differ substantially in the extent to which SDur, timing, and regularity predict mood levels on the following day. We expected substantial heterogeneity of person‐specific associations between sleep patterns and both, sad and anxious mood levels. According to our registered Bayesian approach, substantial heterogeneity among person‐specific associations would be indicated by at least moderate gains its credibility based on informed prior assumptions and the newly observed data.

Our second objective was to investigate if these differences in sleep‐mood dynamics can predict differences in internalizing symptoms reported at the end of the monitoring period.

**FIGURE 1:**
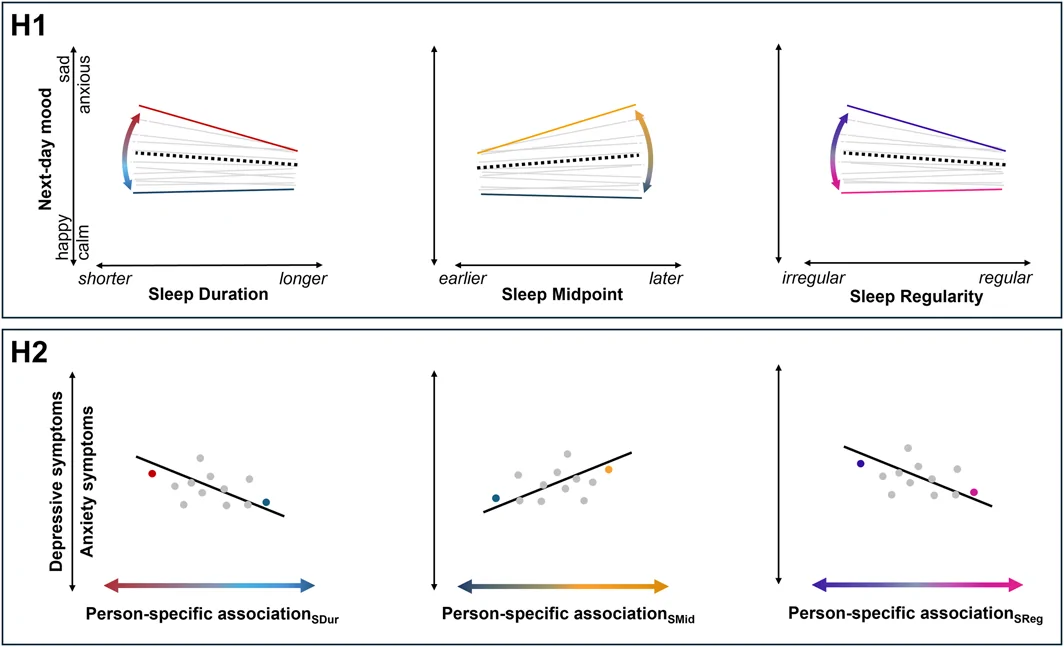
Conceptual illustration of hypothesized relationships among sleep patterns, next‐day mood, and internalizing symptoms. Panel **H1** shows person‐specific associations between daily sleep variations (duration, midpoint, regularity) and next‐day mood. Individual regression lines (i.e., slopes) highlight heterogeneity around the average association (dotted line), with color gradients emphasizing heterogeneity. Panel **H2** depicts corresponding between‐person associations of individual person‐specific associations with depressive and anxiety symptoms. Each regression line from H1 corresponds to one data point in H2 (person‐specific regression coefficients on *x*‐axis; symptom scores on *y*‐axis). Adolescents with stronger person‐specific associations involving worse mood after shorter, later, or irregular sleep are expected to show higher symptom severity. No specific differences between associations involving sad/anxious mood or depressive/anxiety symptoms are hypothesized. SDur = sleep duration; SMid = sleep midpoint; SReg = sleep regularity.

H 2 Adolescents who express stronger person‐specific associations, specifically shorter, later, and less regularly timed sleep periods predicting worse next‐day mood, exhibit higher symptoms of depression and anxiety. Of note, sleep midpoint (SMid) implies an opposite directionality of expected effects than SDur and SReg (see Figure 1). We focused on consistent mood‐symptom pairs aligning associations involving sad mood with depressive symptoms, and those involving anxious mood with anxiety symptoms, respectively. According to our registration, we followed a unidirectional approach where daily fluctuations in sleep patterns predict next‐day mood levels. We chose this approach due to the current limitations in modeling bidirectional dynamics, which require more sophisticated statistical frameworks still under active development (Haslbeck & Ryan, 2022; Murray & Kunicki, 2022; Savord et al., 2023; Usami et al., 2019).

## METHODS

### Methods

The methods and analysis of the present study were co‐registered during an early phase of data collection (Benning et al., 2019; Drexl et al., 2023). Deviations from the registered analysis plan are detailed in the Supporting Information S1 (Appendix S1). Here, we describe the selection of participants, procedures, and methods that apply to the present study. A detailed description of the wider mSanté project is reported elsewhere (Drexl et al., 2025).

### Participants

Adolescent students aged 14–17 were recruited from two French‐speaking and two English‐speaking private schools in Switzerland. Around 330 students were invited through in‐class presentations and school assemblies. Eligibility required: smartphone ownership, fluency in French or English, and absence of significant motor, cognitive, intellectual, or developmental disabilities. These eligibility criteria were transparently communicated at the invitation presentations. Following the presentations, students received additional information letters including the written informed consent forms addressed to them, as well as information letters for their parents. Parents were not required to sign consent forms but were provided with detailed written information about the study, including the invitation to contact the research unit in case of any questions. In line with Swiss law and corresponding regulations, adolescents aged 14 years or older can directly provide written informed consent for participation in studies authorized by ethic committees. They could return signed informed consent forms to the school staff, or the research team up to the respective kick‐off session. Students self‐selected according to eligibility criteria and signed up for grouped enrollment sessions. In total, 113 students provided informed consent. Enrollment occurred between November 2022 and September 2023, with data collection concluding in July 2024.

### Study procedures

Participants were provided with detailed information about how the study was conducted at their school, along with instructions for wearing the actigraphy wristband and training for EMA. Baseline questionnaires were distributed later that same afternoon after the training. Participants were given two to 5 days to complete these baseline measures in their free time before the first of three assessment waves began. Each assessment wave lasted 3 weeks and involved continuous actigraphy and EMA of four prompts per day. In addition, each wave included four weekly assessments of internalizing symptoms. After the final day of each assessment wave, the research team met with participants at school to collect the wristbands and distribute gift vouchers of 30, 30, and 40 Swiss francs, for responding to at least two‐thirds of the EMA prompts of the respective wave. Assessment waves were scheduled to avoid school vacations while also covering a broad range of seasonal variability in environmental exposure to light and temperature. The order of low (winter), moderate (spring and autumn), and high (summer) exposure periods varied across schools according to their respective kick‐off dates (see Supporting Information S1: Appendix S2, Figure S1). Breaks between assessment waves ranged from 40 to 102 days (average of 74.4 days, SD = 16.5). The study concluded with a follow‐up self‐report repeating the general psychopathology scale from the baseline assessment followed by a feedback survey on study acceptability and measurement reactivity. After completing these measures, participants were invited to a group feedback session. All self‐report measures were collected online via REDCap® (Harris et al., 2009, 2019), with links to the online questionnaires sent via SMS or email. Questionnaires were worded in French or English. To stimulate engagement, the questionnaires included illustrations and elements of gamification (Drexl et al., 2025).

### Measures

#### Baseline characteristics

Participants reported age in full years since birth, as required by ethics regulations to strengthen anonymity of participation. The gender item included a third diverse option. For descriptive purposes, we report other variables related to the current family situation, whether the participant was pursuing academic higher education or vocational training, and whether the participant was currently receiving, had ever received, or had never received mental health care. Psychometric scales that entered exploratory analyses are described in the Supporting Information S1.

#### Actigraphy recordings

Throughout each assessment wave, participants wore a triaxial accelerometer (GENEActiv, Activinsights © Ltd.) on their non‐dominant wrist. Recordings were configured at a 30 Hz sampling frequency, beginning the afternoon prior to the first EMA morning delivery. Preprocessing was conducted using the GGIR package version 3.1–3 (Migueles et al., 2019; van Hees et al., 2022) and utilized a heuristic sleep detection algorithm based on arm angle relative to the horizontal plane (van Hees et al., 2015; Van Hees et al., 2018). Detected sleep periods were manually reviewed against raw time‐series data to correct common misclassifications, such as spurious non‐wear periods (Van Hees, 2024; Vert et al., 2022). The preprocessing script for actigraphy data used in this study is available through our open research compendium (https://osf.io/dpsb5).

Preprocessed actigraphy data provided nightly major sleep period duration (in hours) along with sleep onset and wake‐up times. These data enabled the computation of the SMid (Roenneberg et al., 2004) and a modified Sleep Regularity Index (SRI; Phillips et al., 2017). The SMid is the clock time midway between sleep onset and wake‐up. Our SRI calculation followed the procedures of a previous intensive longitudinal study (Vidal Bustamante et al., 2020), expressing the proportional overlap of each 24‐h sleep‐wake cycle with each individual's aggregated most typical sleep‐wake pattern. The resulting metric is sensitive to variability in both SDur and SMid. The maximum of 1 represents perfect overlap with the typical cycle, while a value of −1 indicates complete inversion of sleep‐wake phases.

#### Ecological Momentary Assessment

Participants received four EMA surveys per day over each of the three 3‐week waves. Survey delivery times were fixed to each school's schedule, occurring at four points: before classes (e.g., 7:45 a.m.), during lunch (e.g., 12:00 p.m.), after the last class (e.g., 3:45 p.m.), and in the evening (e.g., 7:30 p.m.). If a participant did not respond, they received two reminders at 20‐min intervals. Surveys remained accessible for up to 3 hours for the first three surveys of the day and 4 hours for the evening survey. Compliance with the EMA protocol was defined as the proportion of completed surveys out of the total number of planned surveys for each assessment wave in which a participant was enrolled. Responses were excluded if they were deemed carelessly quick, when completed in under 30 s, or carelessly extreme, when all items in the mood section were answered uniformly at the minimum or maximum scale value (i.e., 1 or 7).

The present analysis used two items based on the mood circumplex model (Larsen & Diener, 1992). Momentary sad mood was assessed with the question: “How happy versus sad do you feel right now?” (1 = “Very cheerful/happy” to 7 = “Very sad/depressed/unhappy”), and anxious mood with: “How relaxed versus anxious do you feel right now?” (1 = “Very relaxed/calm” to 7 = “Very nervous/anxious”). Daytime levels of sad and anxious mood were calculated as the average of all responses for each participant within a given day. Given the bipolar nature of both items, we will refer to sad‐vs‐happy and anxious‐vs‐calm mood for the remainder of this paper.

#### Depressive symptoms

The Center for Epidemiologic Studies Depression Scale for Children is a 20‐item self‐report questionnaire designed to screen for depressive symptoms experienced in the past week by children and adolescents aged 8–17 years (CES‐DC; Faulstich et al., 1986; Fuhrer & Rouillon, 1989). A recent validation study demonstrated convincing psychometric properties for the combined use of bipolar single items of momentary mood and the adult version of the scale (Cloos et al., 2023). In this study, we modified the original four‐point Likert scale to a six‐point format by dividing the labels at both ends into separate categories (e.g., from 0 = “none of the time or rarely” to 1 = “none of the time” and 2 = “rarely”) to provide more specific and less ambiguous response options. The resulting total scores range from 20 to 120. Across all assessments, the scale yielded an intraclass correlation coefficient of 0.73.

#### Anxiety symptoms

Anxiety symptoms over the past 2 weeks were assessed using the State‐Trait Anxiety Inventory for Children (STAIC; Spiegelberger et al., 1973; Turgeon & Chartrand, 2003). The 20 items of the trait subscale assess the frequency of anxious thoughts and physiological symptoms using a 3‐point Likert scale (1 = “hardly ever,” 2 = “sometimes,” and 3 = “often”), with total scores ranging from 20 to 60. In our sample, 78% of the variance in STAIC scores was attributable to between‐person differences.

### Statistical analyses

Following a 2‐step modeling approach, we first estimated within‐person random‐slopes of sleep patterns and next‐day mood levels, which were then used as predictors in Step 2 to predict corresponding internalizing symptoms. We conducted six separate model sets by combining each mood outcome (sad or anxious) with three sleep metrics (duration, midpoint, and regularity).

For the Step‐1 within‐person models, we included all available day‐level data from participants who provided at least 6 sequences of mood‐sleep‐mood observations. Step‐2 between‐person models additionally required presence of the initial and last assessments of depressive or anxiety symptoms, respectively. Step‐1 models included random intercepts and slopes. We calculated person‐specific associations as the sum of the fixed slope and individual random slope estimates.

Sleep duration and SMid were person‐mean centered and combined with person means as covariates to isolate within‐person variation, while the modified SRI readily expresses within‐person variation. To prevent downward bias in predictor estimates, we followed Hamaker & Grasman (2015) and did not center the mood outcome variable. Additional Step‐1 covariates included age, gender, previous‐day mood levels, and current wave number, based on similar analyses (Shen et al., 2022; Vidal Bustamante et al., 2020) and causal diagram analysis (Textor & Liskiewicz, 2012). Posterior distributions resulted from four chains each providing 1000 warm‐up, and 3000 included Markov‐Chain‐Monte‐Carlo (MCMC) draws to obtain Bayesian Credible Intervals (CrI). To express evidence for the presence of individual differences in random slopes (Step 1), we calculated the inverse Bayes Factor BF_10_ of the point‐null BF_01_. We interpreted evidence as absent (BF = 1), anecdotal (BF ≥ 1), moderate (BF ≥ 3), strong (BF ≥ 10), very strong (BF ≥ 30), or extreme (BF ≥ 100), with respective inverted levels in favor of the null‐hypotheses, as per Lee & Wagenmakers (Lee & Wagenmakers, 2014).

Step‐2 models were performed, when Step‐1 models received at least moderate evidence (BF_10_ > 3) in favor of heterogeneity of among person‐specific associations. The registered adjustment set included the respective random intercepts and baseline symptom severity. Based on previous frequentist work (Dzubur et al., 2020), we accounted for the uncertainty in individual slope estimates, by resampling 1000 parameter draws from the effective 12,000 Step‐1 posterior samples, estimated the respective Step‐2 model on each random draw with four chains of 2000 iterations, including 500 for warmup per parameter draw. Model parameters and CrIs were then pooled from the 1000 posterior distributions. Based on the formulation of our registered between‐person hypothesis, we calculated the BF_10_ for the regression coefficients of person‐specific associations acting as the predictor.

For both model steps, we registered informative priors inspired by previous research (see Supporting Information S1: Appendix S3, for model equations and priors). Model convergence was assessed based on MCMC sampling diagnostics, specifically R‐hat values below 1.1 (Gelman, 2006) and effective sample sizes (ESS) of at least 1000 draws (Bürkner, 2017). Influential observations were identified using k‐values above 0.7 via Pareto‐smoothed importance sampling for leave‐one‐out cross‐validation (Vehtari et al., 2017, 2022). For multilevel models, we report both conditional and marginal Bayesian *R* ^2^.

Sensitivity analyses tested the influence of different prior distributions on the BF_10_ for the focal model parameters and are detailed in the Supporting Information S1 also presenting the registered exploratory analyses, including various sensitivity checks, model extensions, and alternative sleep indicators. All statistical analyses were carried out in R version 4.3.0 (R Core Team, 2023) and Bayesian models were estimated with the brms package (Bürkner, 2017). The supporting data set and statistical code are accessible via Open Science Framework repository (https://osf.io/dpsb5).

## RESULTS

### Data availability

**FIGURE 2:**
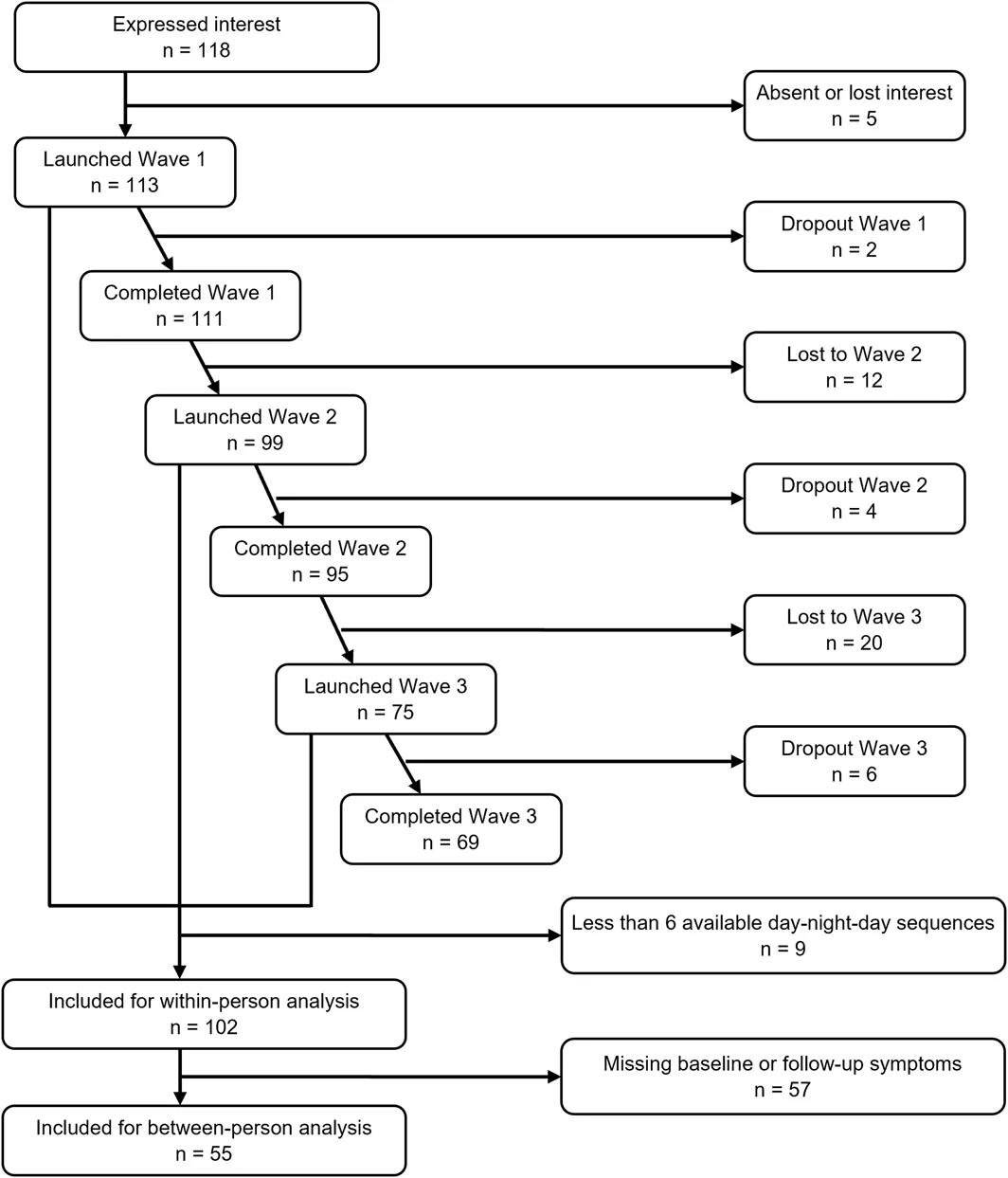
Participant flow chart of the multi‐wave intensive longitudinal protocol. Considered for analysis were all participants who provided at least 6 sequences of day‐night‐day assessments irrespective of having completed the third wave.

Of the 113 participants who enrolled in the protocol, 44 (39%) discontinued, mostly before the respective subsequent assessment wave began (Figure 2). Communicated reports of dropout reasons included perceptions that the protocol was too time‐consuming (*n* = 10), external factors like leaving school (*n* = 6), or parental opposition (*n* = 2). Whether the latter reflected a strict parental decision, or a joint family decision was not explored, as the right to withdraw from participation had to remain unconditional and voluntary. Three participants enrolled at a later wave compared to their peers because they were still 13 years old when the first wave started at their school. The average EMA compliance rate was 73.83% (SD = 22.22%) for participants who responded at least once during an assessment wave. On average, participants provided valid sleep recordings for 41.9 nights (SD = 19.08) each. Approximately half of the participants completed both initial and final symptom assessments (CES‐D: *n* = 55, 49.1%; STAIC: *n* = 53, 47.3%). After crossing all data sources, we retained 4087 days‐night‐day sequences involving sad‐vs‐happy mood (60.0%) and 4004 sequences involving anxious‐vs‐calm mood (58.7%; Supporting Information S1: Figures S2.1–S2.6, and Appendix S4, Figure S3). Compliance was unrelated to baseline symptoms, daytime mood, sleep metrics, age, and gender (*r*s < 0.15, *p*s > 0.05; Supporting Information S1: Figure S4).

### Descriptive characteristics

**FIGURE 3:**
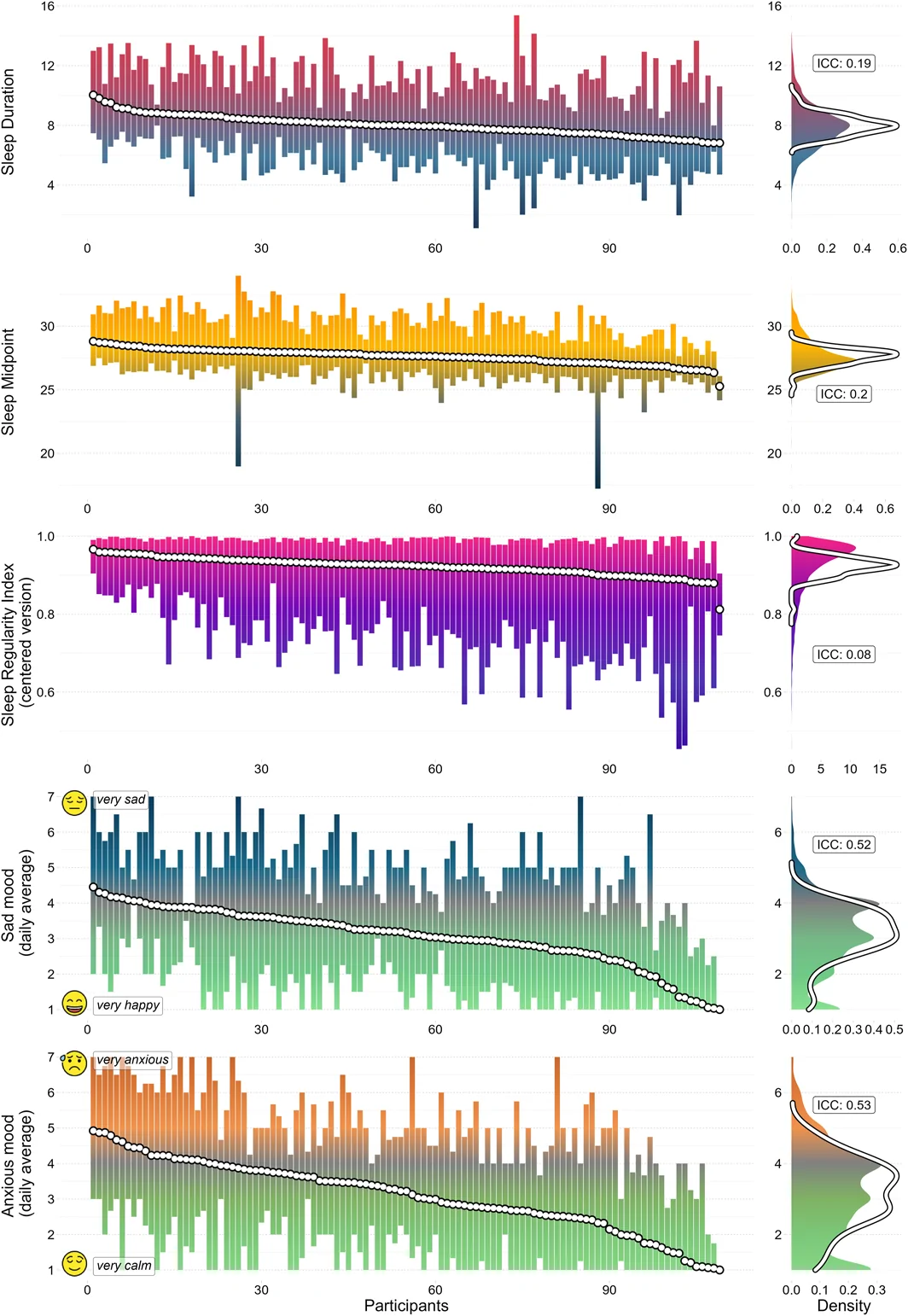
Within and between‐person distributions of sleep metrics and daily average mood levels. The vertical bars depict the full range of each participant colored relative to the position on the *y*‐axis. Participants appear in descending order of the respective person mean (white dots). Emojis and corresponding labels were originally used as anchors on the item response scale. The right margin depicts the densities of the within and between‐person data space. The intraclass correlation coefficient quantifies the proportion of between‐person variance over the total variance observed in a given variable. Emojis designed by OpenMoji—the open‐source emoji and icon project. License: CC BY‐SA 4.0.

The majority of participants was female (Table 1), self‐identified as white, and was primarily anglophone. The average age was 15.04 years (SD = 0.97; Table 1). Sleep and mood variables showed varying degrees of within‐person and between‐person variance (Figure 3). Most sleep metrics exhibited a strong within‐person component, whereas variance of daily mood levels was mostly attributed to between‐person differences. Pairwise day‐level associations indicated that participants slept longer, later, and more irregularly on weekends than during the week, while daytime mood remained stable (Supporting Information S1: Figures S4 and S5).

**TABLE 1:** Sample characteristics overall and by inclusion in symptom prediction models (Step 2).

Characteristic	N	Overall (*N* = 115^a^)	Included (*N* = 55^a^)	Excluded (*N* = 60^a^)	*p*‐value^b^
Females	112	64 (57%)	36 (65%)	28 (49%)	0.081
Age	112	15.04 (0.97)	15.05 (1.01)	15.04 (0.94)	>0.9
Non‐white	112	23 (21%)	8 (15%)	15 (26%)	0.12
Nationality	111				0.3
Swiss		43 (39%)	24 (44%)	19 (34%)	
Other		68 (61%)	31 (56%)	37 (66%)	
Language	115				0.007**
French		20 (17%)	15 (27%)	5 (8.3%)	
English		95 (83%)	40 (73%)	55 (92%)	
Separate parent households	110	21 (19%)	11 (21%)	10 (18%)	0.7
School track	111				0.5
Vocational		26 (23%)	11 (20%)	15 (26%)	
Academic		85 (77%)	43 (80%)	42 (74%)	
Receiving mental health care	112				0.011^d^
Current		19 (17%)	14 (25%)	5 (8.8%)	
Lifetime		17 (15%)	11 (20%)	6 (11%)	
Never		76 (68%)	30 (55%)	46 (81%)	
Sleep duration	111	8.01 (0.67)	7.97 (0.64)	8.05 (0.70)	0.8
Sleep midpoint^b^	111	27.61 (0.60)	27.64 (0.59)	27.57 (0.60)	0.8
Sad mood	113	3.05 (0.80)	3.08 (0.75)	3.02 (0.85)	0.8
Anxious mood	113	3.09 (0.97)	3.10 (0.97)	3.08 (0.98)	>0.9
CES‐DC^c^ (6‐point likert)	104	56 (16)	58 (15)	55 (17)	0.2
STAIC^c^	99	39 (8)	40 (8)	38 (8)	0.4

*Note*: Inclusion in symptom prediction models depended essentially on availability of baseline and follow‐up symptom severity scores. The included group represents the union set of participants entering either the depressive symptom model or the anxiety symptom model.

Abbreviations: CES‐DC, Center for Epidemiological Studies—Depression Scale for Children; STAIC, State Trait Anxiety Inventory for Children.

^a^ *n* (%); Mean (SD).

^b^ In hours following midnight of the previous day. For example, a sleep midpoint of 27 corresponds to a major sleep period centered around to 3:00 am.

^c^ Averaged across available weekly assessments.

^d^ *p < 0.05; **p < 0.01; ***p < 0.001; *t*‐test; Fisher's *z*‐test.

### Person‐specific associations between sleep and daytime mood

**FIGURE 4:**
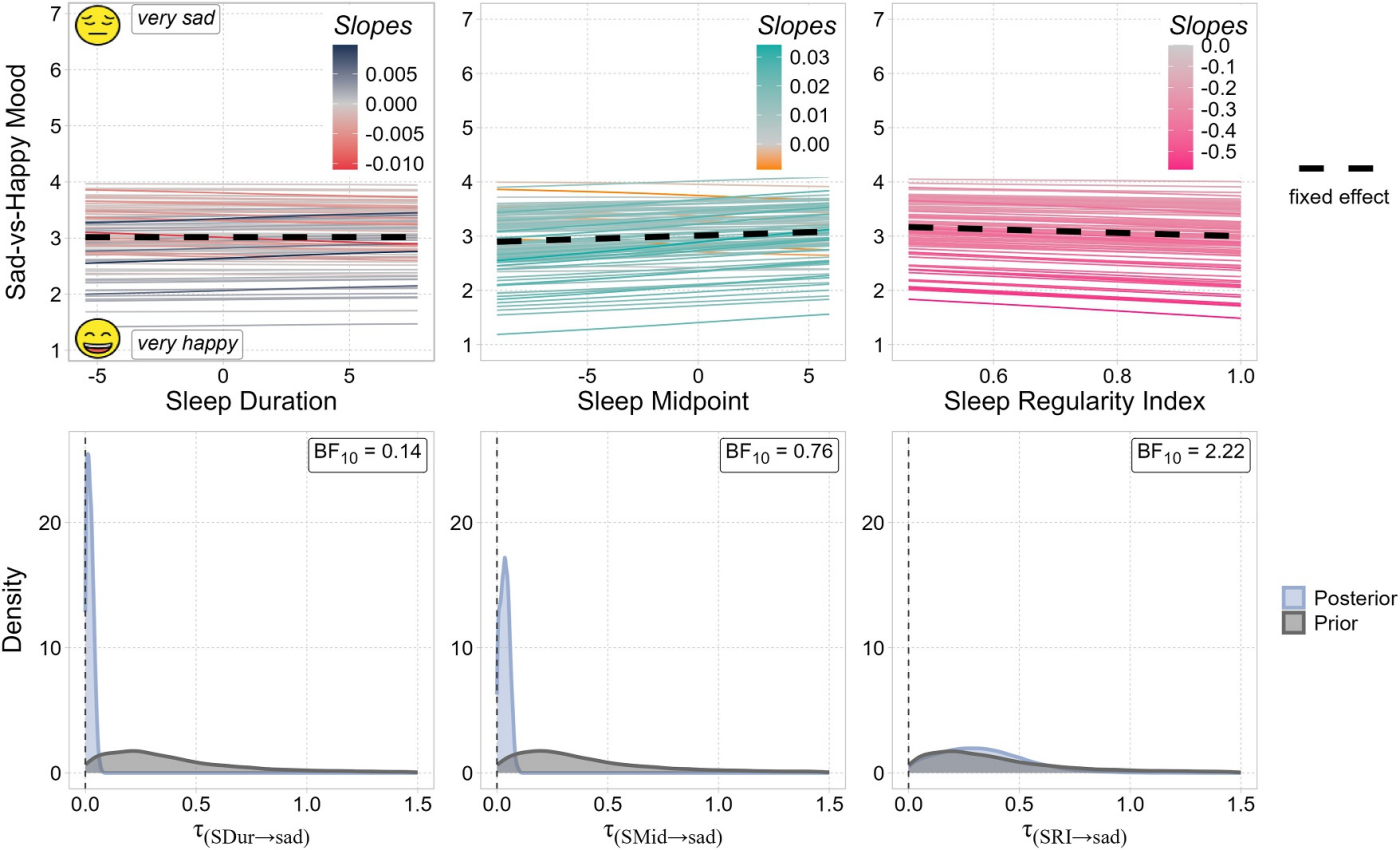
Person‐specific associations between sleep patterns and sad‐vs‐happy mood. The top row panels predicted mood‐levels for each participant per sleep pattern. Each slope represents an individual person‐specific associations and is colored as a function of its angle. The bottom row panels compare the corresponding prior and the posterior distributions for the standard deviation of random slopes. Bayes Factors (BF_10_) quantify evidence for the standard deviation being greater than zero. Labeled emoticons correspond to the scale anchors of the respective seven‐point Likert item. Emojis designed by OpenMoji—the open‐source emoji and icon project. License: CC BY‐SA 4.0. SDur, sleep duration; SMid, sleep midpoint; SRI, Sleep Regularity Index.

Our analyses at the within‐person level (Step 1) revealed no substantial evidence in favor of heterogeneity in person‐specific associations between SDur and sad‐vs‐happy mood (BF_10_ = 0.14, see Table 2 and Figure 4; full model results are presented in Supporting Information S1: Appendix S5, Tables S1.1–S1.3). For person‐specific associations between SMid and sad‐vs‐happy mood, the model showed moderate evidence for a small positive fixed slope (*b* = 0.01, 95%CrI [−0.02 to 0.04], BF_10_ = 3.45), but no evidence in favor of variation around this general effect (BF_10_ = 0.76). Sleep regularity and sad‐vs‐happy mood received strong evidence for a positive fixed‐slope (*b* = −0.33, 95%CrI [−0.71 to 0.08], BF_10_ = 12.94) and anecdotal evidence for variation of individual effects (*τ* ^2^ = 0.33; 95%CrI [0.03–0.87], BF_10_ = 2.22).

**TABLE 2:** Fixed slopes and variation in random slopes.

Model	Fixed slope			Random slopes			*R* ^2^_c_/*R* ^2^_m_
	B	95% CrI	BF_10_	*τ* ^2^	95% CrI	BF_10_	
Sad‐vs‐happy (*N* = 4099)							
Sleep duration	<0.01	−0.02 to 0.02	1.30	0.02	<0.01–0.06	0.14	0.54/0.17
Sleep midpoint	0.01	−0.02–0.04	3.45	0.04	<0.01–0.08	0.76	0.54/0.18
Sleep regularity	0.33	−0.71 to 0.10	12.94	0.33	0.03–0.87	2.22	0.54/0.14
Anxious‐vs‐calm (*N* = 4013)							
Sleep duration	−0.03	−0.05 to −0.01	130.9	0.04	<0.01–0.07	0.83	0.59/0.20
Sleep midpoint	0.01	−0.02–0.04	1.17	0.04	<0.01–0.09	0.49	0.59/0.19
Sleep regularity	−0.26	−0.70 to 0.20	8.02	0.47	0.09–0.79	13.2	0.59/0.18

*Note*: Bayes Factors for fixed slopes refer to evidence in favor of greater sleep duration, earlier sleep midpoint, and a higher SRI predicting more happy or calm next‐day mood, respectively. With better mood expressed by lower values of the bipolar scale, these directional effects correspond to negative slopes for duration and regularity, and positive slopes for sleep midpoint. Covariates included age, gender, number of assessment wave, weekend, previous day mood, and person‐averaged sleep metric except for sleep regularity which is already self‐referential by calculation. According to Lee & Wagenmakers (Lee & Wagenmakers, 2014), levels of evidence were defined a priori as absent (BF = 1), anecdotal (BF ≥ 1), moderate (BF ≥ 3), strong (BF ≥ 10), very strong (BF ≥ 30), or extreme (BF ≥ 100), with respective inverted levels in favor of the null‐hypotheses.

Abbreviations: 95% CrI, 95% Posterior Credible Interval; BF_10_, Bayes factor; *R* ^2^_c_, Bayesian conditional; *R* ^2^_m_, Bayesian marginal *R* ^2^.

**FIGURE 5:**
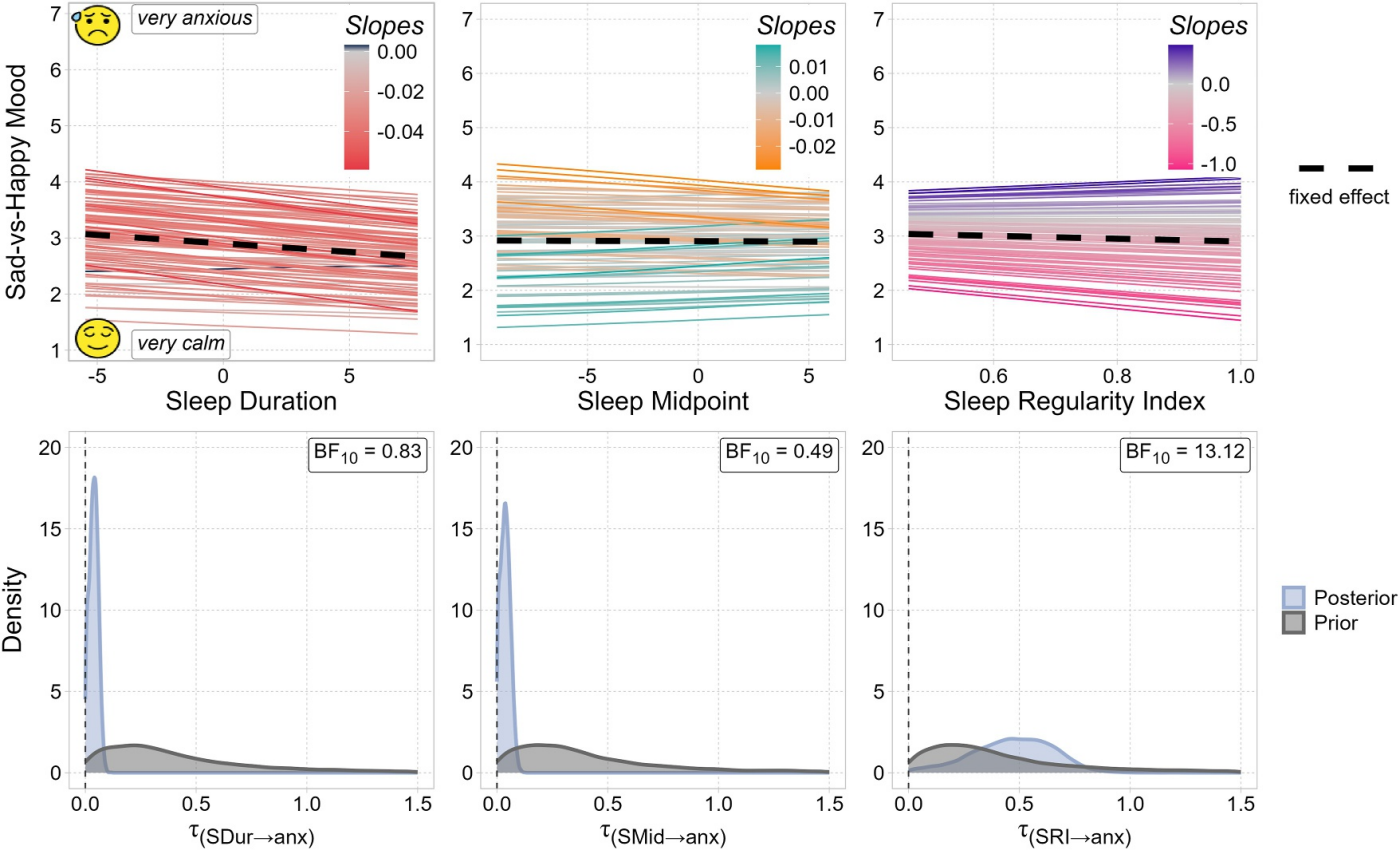
Person‐specific associations of sleep patterns and anxious‐vs‐calm mood. The top row panels predicted mood‐levels for each participant per sleep pattern. Each slope represents a person‐specific association and is colored as a function of its angle. The bottom row panels compare the corresponding prior and the posterior distributions for the standard deviation of random slopes. Bayes Factors (BF_10_) quantify evidence for the standard deviation being greater than zero. Labeled emoticons correspond to the scale anchors of the respective seven‐point Likert item. Emojis designed by OpenMoji—the open‐source emoji and icon project. License: CC BY‐SA 4.0. anx, anxious mood; SDur, sleep duration; SMid, sleep midpoint; SRI, Sleep Regularity Index.

For person‐specific associations between SDur and anxious‐vs‐calm mood, we found extreme evidence for a negative fixed effect (*b* = −0.03, 95%CrI [−0.05 to −0.01], BF_10_ = 130.87, see Figure 5), but no evidence for individual differences (BF_10_ = 0.83). Sleep midpoint and anxious‐vs‐calm mood showed neither a general effect, nor individual differences in person‐specific associations (BF_10_ = 0.49). Finally, results showed strong evidence for individual differences in person‐specific associations of SReg and anxious‐vs‐calm mood (*τ* ^2^ = 0.47, 95%CrI [0.09; 0.79], BF_10_ = 13.2). The most extreme person‐specific associations marked a change of 0.11 points toward calm mood versus a modest change of 0.05 points toward anxious mood, both per 0.1 improvement in SReg. The following section focuses on whether individual differences in person‐specific associations between anxious‐vs‐calm mood and SReg predict anxiety symptoms. Since the other types of person‐specific associations did not show evidence for interindividual differences, interpreting corresponding predictions for depressive symptoms is not warranted. For completeness, these estimates are reported in the Supporting Information S1 (Tables S2.1–S2.3).

### Linking person‐specific associations between sleep‐regularity and anxious‐vs‐calm mood with symptoms of anxiety

**FIGURE 6:**
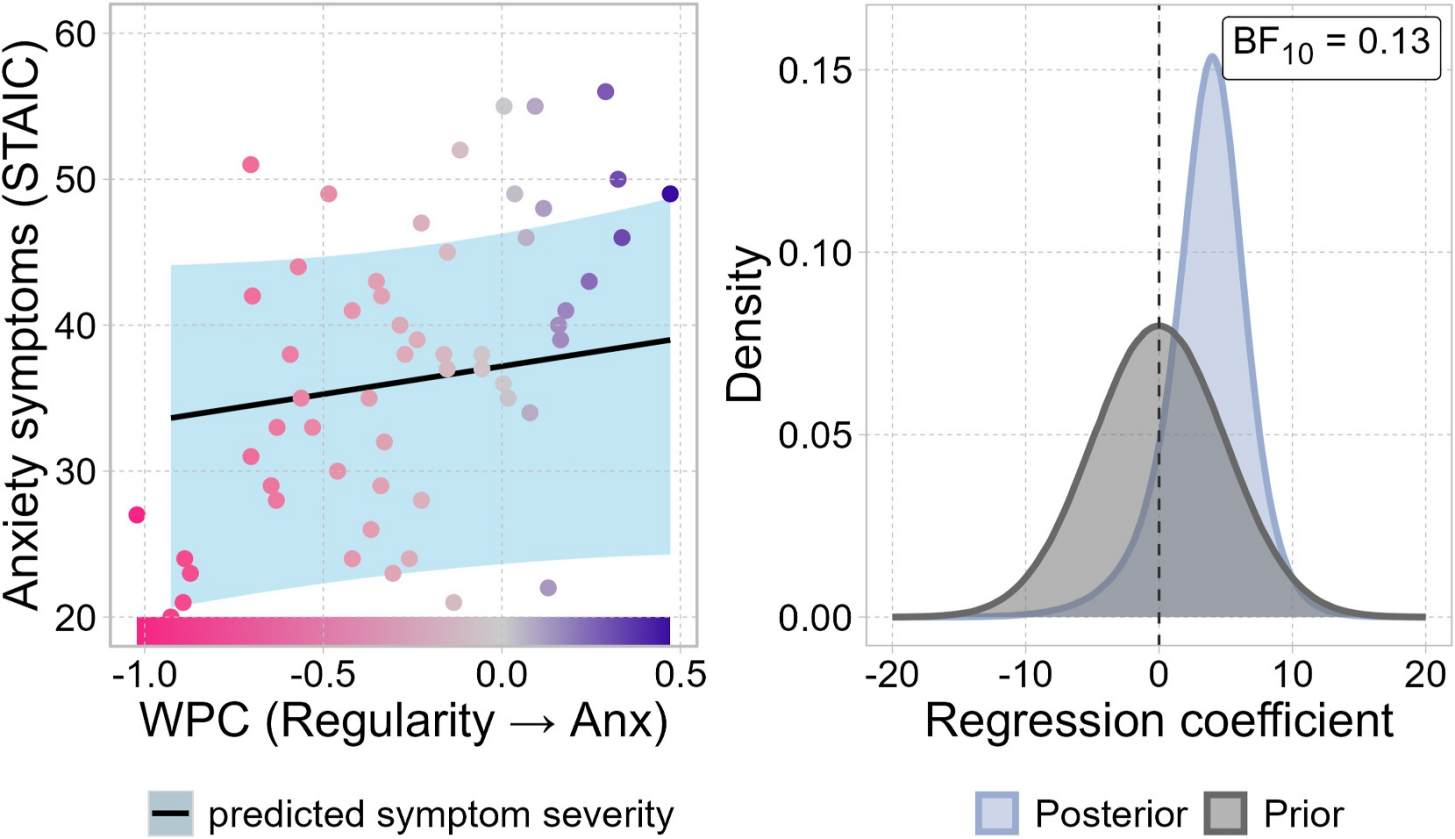
Prediction of anxiety symptoms by person‐specific associations between sleep regularity (SReg) and anxious‐vs‐calm mood. Person‐specific associations, that is, slopes from the Step‐1 models, are plotted on the *x*‐axis of the left panel with the corresponding color gradient as in Figure 5. The black solid line shows the pooled linear effect when controlling for baseline symptoms and random intercepts. The right panel compares the corresponding prior and the posterior distributions of the regression coefficient. Anx, Anxious‐vs‐calm mood. BF_10_, Bayes Factor for the regression coefficient being smaller than zero.

We found moderate evidence for the association between symptoms of anxiety and differences in person‐specific associations between SReg and anxious‐vs‐calm mood (*b* = 3.57, 95%CrI [‐3.67; 9.31], BF_01_ = 7.69, *R* ^2^ = 0.44; Figure 6). Adolescents whose calm mood was most strongly linked to having regular sleep–wake cycles reported the lowest levels of anxiety symptoms at the end of the monitoring period, after adjusting for baseline anxiety. These adolescents also showed the strongest overall person‐specific associations in absolute terms. In contrast, the rest of the sample displayed either weaker associations in the same direction, essentially uncoupled (i.e., close to zero) relations, or reversed but still weaker associations (see Figures 5 and 6). Consequently, the highest levels of anxiety symptoms were observed among adolescents who tended to feel more anxious after more regular nights of sleep. However, this counterintuitive dynamic was less pronounced than the dynamic of regularity predicting calm mood seen in adolescents with the lowest levels of anxiety. Finally, the present sample could not demonstrate whether feeling comparatively more anxious after irregular sleep–wake cycles predicted more intense anxiety symptoms, as the predicted mood levels only marginally extended into the anxious dimension of the anxious–vs–calm spectrum (see Figure 5).

Furthermore, we detected strong collinearity between random intercepts and the person‐specific association predictor, as indicated by an excessively high correlation between random intercepts and random slopes (*p* = 0.98; see Supporting Information S1: Table S3). Dropping the random intercept covariate increased the magnitude of the association, as well as its level of evidence (*b* = 4.34, 95%CrI [‐2.98; 10.52], BF_01_ = 10.96). Results based on the originally registered target dataset are presented in the Supporting Information S1 (Appendix S6, Tables S4 and S5).

### Sensitivity checks and exploratory analyses

Further sensitivity analyses indicated that Bayes Factors for the heterogeneity in person‐specific associations were to some extent sensitive to prior specifications. Stronger priors showed decisive evidence for the presence of differences in associations involving SMid and sad‐vs‐happy mood (Supporting Information S1: Appendix S7, Figures S6 and S7). Removal of one participant who exhibited high influence on model fit (Pareto *k* > 0.7, Supporting Information S1: Figures S8 and S9) affected only two between‐person models and showed no change in evidence for either side (Supporting Information S1: Table S6). Data availability, quantified as the number of analyzed data‐points per person was unrelated to person‐specific associations between SReg and anxious mood (*ρ* = −0.06, 95%CrI [−0.24, 0.14], BF_10_ = 0.28). The Supporting Information further presents model results based on explicit person‐mean centering of SRI scores (Supporting Information S1: Table S7) and collinearity among predictors in the model predicting anxiety by SRI‐related couplings (Supporting Information S1: Table S3). With respect to the great impact on analytical attrition that our registered decision to use only the last weekly symptom report as the between‐person outcome, we performed an additional sensitivity check based on a more inclusive model that uses the average of all available STAIC reports as the outcome predicted by person‐specific associations between SReg and anxious‐vs‐happy mood. This model included 96 participants (84.9%) and yielded the same directional finding (*B* = 6.44; 95% CI 1.51–11.39; BF_01_ = 63.52; see Supporting Information S1: Table S8). The increased level of evidence indicates that the pattern is not driven by selective inclusion for analyses but that the smaller registered selection criteria primarily reduced precision (i.e., wider uncertainty) rather than shifting the effect. Analytic sample size and level of evidence did not improve when using a generic internalizing score as person‐level outcome (Supporting Information S1: Table S9).

Results of registered exploratory analyses are presented in the Supporting Information (Supporting Information S1: Appendix S8, Tables S10.1–S18, Figures S10 and S11). Noteworthy results include extreme evidence for the presence (B_10_ = 8.59 × 10^14^) of individual differences in person‐specific associations between subjective sleep quality and sad‐vs‐happy mood and moderate evidence in favor or individual differences among associations between sleep quality and anxious mood (BF_10_ = 3.67; Supporting Information S1: Table S13).

## DISCUSSION

### Summary of key findings

This study examined individual differences in person‐specific associations between sleep and mood among adolescents using intensive longitudinal data with up to 63 days of actigraphy and EMA per participant. Sleep duration and midpoint presented no or less evident individual differences in their association with daytime mood but revealed distinct group‐level effects with shorter SDur predicting changes toward sad mood and later SMid predicting changes toward anxious mood. However, our first hypothesis was partially confirmed as the present sample provided strong evidence that adolescents' anxious‐vs‐calm mood does not uniformly react to daily fluctuations of the sleep‐wake cycle. Adolescents whose calm mood was more strongly linked to having a regular sleep‐wake cycle showed fewer symptoms of anxiety at the end of the monitoring period, as indicated by moderate evidence in favor of a linear relationship after controlling for baseline symptoms and person‐specific average mood levels (i.e., random‐intercepts). This is inconsistent with our second hypothesis (H2) expecting stronger person‐specific associations between poorer sleep and negative mood to be linked with internalizing symptoms. With respect to the collinearity of overall mood levels and these person‐specific associations, the present findings suggest that having calm mood more tightly connected to the with the preceding sleep‐wake cycle is characteristic for adolescents with overall calm mood as well as attenuated levels of anxiety symptoms at follow‐up. Adolescents with higher anxiety severity, by contrast, showed weaker, absent, or counterintuitive but still weaker sleep‐mood associations, with some adolescents experiencing marginal increases in anxious more following more regular sleep‐wake cycles. This requires further investigation. Taken together, this study provides the first illustration of how the sleep‐wake cycle is dynamically linked with affective daytime functioning in adolescents and how certain person‐specific sleep–mood associations may reflect protective dynamics against emerging internalizing symptoms. While our findings contribute to a more fine‐grained and idiographic understanding of the sleep‐mood interface, this approach also introduces complex interpretations that warrant some conceptual clarification.

### Interpreting person‐specific associations between sleep and mood on the daily level

To correctly interpret the direction and strength of person‐specific associations between sleep and mood, we must consider both the characteristics of the mood scales and those of the specific sleep metrics used. In this study, we assessed mood using symmetric, or bipolar, scales, where high values represented negative moods and low values represented positive ones. Importantly, fluctuations in either direction are not inherently “good or bad”. Our data showed that mood levels were often positive or neutral, making it challenging to draw robust conclusions for the hypothesized heterogeneity in dynamics between sleep and mood.

It is also important to acknowledge that SDur and SMid often overlap in natural settings. Later bedtimes typically result in shorter sleep durations during the school week. Given this interconnected nature of sleep metrics, the modified SReg Index (Vidal Bustamante et al., 2020) emerged as the most sensitive indicator for detecting differences in person‐specific associations between sleep and mood, likely because it captures fluctuations in both SDur and SMid and thereby reflects behavioral manifestations of circadian stability or disruption. In real‐life adolescent routines, irregularity often arises from conflicts between biological sleep needs and social demands, resulting in circadian misalignment (Crowley et al., 2018). Such circadian disturbances may therefore help explain why SReg plays a particularly important role in mood regulation. Further research is needed to disentangle the specific contributions of SDur, SMid, and circadian functioning to affective processes. At the same time, it is essential to recognize that person‐specific associations should not be understood as deterministic causal mechanisms. Instead, they represent temporal signatures of broader biopsychosocial processes (Blake, Trinder, & Allen, 2018), including neurobiological systems (e.g., circadian regulation, neurotransmitter activity) and contextual influences such as academic or social stressors, which may jointly shape sleep, mood, and psychiatric symptoms.

### Comparison with previous work

Previous research has emphasized the negative effects of poor sleep on daytime affective functioning, with support from a broad experimental evidence base (Palmer et al., 2024; Tomaso et al., 2021). In contrast, observational research presents greater inconsistencies (Hickman et al., 2024). The group‐level results found in our within‐person models align partly with this literature, suggesting modest average benefits in positive, low‐arousal mood (calm) with increased SDur (Shen et al., 2022). Observational studies examining daily associations involving indicators of SMid and SReg are scarce, but our average within‐person effects extend recent cross‐sectional and prospective analyses of this metric in adolescents (Castiglione‐Fontanellaz et al., 2023; Lemke et al., 2023; Yan et al., 2024).

However, this study revealed clear differences in daily person‐specific sleep–mood associations among adolescents, irrespective of the average within‐person effect. These findings shed new light on the previous literature, especially because we demonstrated that individual deviations from a Null‐effect at the group‐level may indeed be related to symptom severity. Considering that the spectrum of person‐specific effects ranged from stronger, to weaker, absent, and even opposite relations within the present sample, it is not surprising that previous group‐level effects findings resulted in large inconsistencies across different samples as a consequence of sampling variability (Hickman et al., 2024). Moreover, linking these individual dynamics with between‐person outcomes of symptom severity opens new opportunities to investigate how sensitivity to fluctuations in sleep patterns may influence risk and resilience in emerging internalizing disorders (Becker et al., 2017; Berkhout et al., 2025; Blake, Trinder, & Allen, 2018).

### Limitations

While our intensive longitudinal design allowed a more idiographic understanding of adolescents' person‐specific sleep–mood associations, several limitations must be addressed. First, the homogeneous nature of our sample, which included only adolescents from private schools in Switzerland, may have reduced heterogeneity in person‐specific associations. It also limits the generalizability of our findings to more diverse populations, which may be more affected by sleep disturbances (El‐Sheikh et al., 2022; Giddens et al., 2022). Second, we did not conduct comprehensive diagnostic assessments for internalizing and sleep disorders, which restricts our ability to draw conclusions that relate to clinical levels of these pathologies. Diagnostic interviews could also have helped elucidating whether the registered analytical dataset was clinically unrepresentative, given that it showed a higher proportion of participants currently receiving mental health care. Moreover, diagnostic eligibility evaluations would have eradicated any uncertainty that adolescents incorrectly self‐selected into the study. Third, our focus on SDur, midpoint, and regularity, and some alternative metrics addressed by exploratory analyses, does not encompass all aspects of sleep (Buysse, 2014). Incorporating additional measures like sleep efficiency and sleep onset latency could yield a more detailed understanding of sleep‐mood dynamics. Fourth, our examination provided limited control for contextual factors like differences in individual week schedules. Thus, weekdays with late first classes or weekend days with exceptional early morning appointments may have added noise to data that remained unaddressed. Lastly, our registered longitudinal analysis focusing on end‐of‐study outcomes, although insightful, was vulnerable to dropout, which affected our ability to draw robust conclusions at the between‐person level. Future studies should consider strategies to enhance participant retention, such as reducing the participant burden or increasing incentives.

### Implications for future research and practice

Our findings provide initial indication that within‐person dynamics between sleep and next‐day mood varies significantly in direction and strength among adolescents. Instead of attempting to identify a nomothetic overall association, future research should investigate the contexts and conditions that shape the interactions between sleep and mood at the individual level. Again, this is especially important, because we consider the person‐specific associations to reveal only the temporal signature at the surface emitted by the underlying more complex mechanisms. Targeting these mechanisms includes examining biological, psychosocial, and environmental factors that may amplify these detectable person‐specific associations, for instance, by comparing periods with circadian pressure to periods of unrestricted sleep (Shen et al., 2022). Given the modest effect sizes predicted for most adolescents in our sample, we suggest exploring differences in person‐specific associations at broader timescales, ranging from weeks to months (Vidal Bustamante et al., 2020). This approach could bridge the gap between existing daily‐level studies (Hickman et al., 2024) and long‐term longitudinal research (Bauducco et al., 2024), while also allowing assessment of clinically meaningful indicators such as accumulated sleep debt and circadian misalignment. Additionally, integrating our intraindividual perspective could help future research better understand the dynamic reciprocal effects of sleep and different forms of affective daytime functioning, such as reactive emotions and emotion regulation (Haslbeck & Ryan, 2022; Lollies et al., 2022). Examining the diversity of these person‐specific associations in heterogeneous adolescent populations including those with internalizing symptoms and sleep disorders, remains a key area of focus.

The intraindividual perspective may also prove valuable in clinical applications, provided future research establishes consistent associations with meaningful effect sizes. Targeted prevention could then involve monitoring sleep and daytime mood in tandem as a strategy to detect heightened reactivity to sleep disturbances, thereby identifying adolescents at increased risk (Blake & Allen, 2020). Finally, assessing temporal links between sleep and mood during sleep‐based interventions for internalizing disorders could help determine which individuals benefit most from treatment (Blake, Blake, et al., 2018; Boland et al., 2023).

## CONCLUSION

In conclusion, the present study provides preliminary indication that adolescents exhibit distinct associations between SReg and next‐day anxious‐vs‐calm mood. Results also indicated adolescents whose calm mood was more tightly coupled with regular sleep‐wake cycles predicted lowest levels of anxiety at the end of the monitoring period, suggesting potential protective effects of regular sleep schedules. These findings emphasize the importance of an idiographic understanding of sleep and mood dynamics in adolescents, though further research in heterogeneous populations is necessary to fully understand the complexity of the individual sleep‐mood interplay and its contribution to emerging internalizing disorders. Ultimately, this ideographic approach may inform personalized approaches for risk, resilience, and sleep‐based interventions for internalizing disorders.

## AUTHOR CONTRIBUTIONS

**Konstantin Drexl**: Conceptualization; investigation; writing—original draft; methodology; visualization; software; formal analysis; data curation; validation. **Setareh Ranjbar**: Supervision; conceptualization; methodology; writing—original draft. **Sébastien Urben**: Conceptualization; supervision; methodology; writing—original draft. **Kerstin Jessica Plessen**: Conceptualization; writing— original draft; supervision; resources; project administration. **Jennifer Glaus**: Project administration; supervision; methodology; conceptualization; investigation; writing—original draft.

## CONFLICT OF INTEREST STATEMENT

The authors declare no conflicts of interest.

## ETHICAL CONSIDERATIONS

All participants included in this study provided written informed consent. The procedures of the present study were approved by the Ethics Committee of the Canton of Vaud (CER‐VD, Project ID: 2021‐01821, April 19, 2022).

## Supporting information

Supporting Information S1

## Data Availability

The data that support the findings of this study are openly available in the Open Science Framework at http://doi.org/10.17605/OSF.IO/YQA49, reference number: yqa49.

## References

[jcv270082-bib-0001] Alfano, C. A. , Pina, A. A. , Zerr, A. A. , & Villalta, I. K. (2010). Pre‐sleep arousal and sleep problems of anxiety‐disordered youth. Child Psychiatry and Human Development, 41(2), 156–167. 10.1007/s10578-009-0158-5 19680805 PMC2818382

[jcv270082-bib-0002] Alvaro, P. K. , Roberts, R. M. , & Harris, J. K. (2013). A systematic review assessing bidirectionality between sleep disturbances, anxiety, and depression. Sleep, 36(7), 1059–1068. 10.5665/sleep.2810 23814343 PMC3669059

[jcv270082-bib-0003] Bauducco, S. , Gardner, L. A. , Smout, S. , Champion, K. E. , Chapman, C. , Gamble, A. , Teesson, M. , Gradisar, M. , & Newton, N. C. (2024). Adolescents’ trajectories of depression and anxiety symptoms prior to and during the COVID‐19 pandemic and their association with healthy sleep patterns. Scientific Reports, 14(1), 10764. 10.1038/s41598-024-60974-y 38730014 PMC11087504

[jcv270082-bib-0004] Becker, S. P. , Sidol, C. A. , Van Dyk, T. R. , Epstein, J. N. , & Beebe, D. W. (2017). Intraindividual variability of sleep/wake patterns in relation to child and adolescent functioning: A systematic review. Sleep Medicine Reviews, 34, 94–121. 10.1016/j.smrv.2016.07.004 27818086 PMC5253125

[jcv270082-bib-0005] Bei, B. , Manber, R. , Allen, N. B. , Trinder, J. , & Wiley, J. F. (2017). Too long, too short, or too variable? Sleep intraindividual variability and its associations with perceived sleep quality and mood in adolescents during naturalistically unconstrained sleep. Sleep, 40(2), zsw067. 10.1093/sleep/zsw067 28364491

[jcv270082-bib-0006] Benning, S. D. , Bachrach, R. L. , Smith, E. A. , Freeman, A. J. , & Wright, A. G. C. (2019). The registration continuum in clinical science: A guide toward transparent practices. Journal of Abnormal Psychology, 128(6), 528–540. 10.1037/abn0000451 31368732 PMC6677163

[jcv270082-bib-0007] Berkhout, S. W. , Schuurman, N. K. , & Hamaker, E. (2025). How to model ambulatory assessments measured at different frequencies: An N=1 approach. OSF. 10.31234/osf.io/6e9w7 40932144

[jcv270082-bib-0008] Blake, M. J. , & Allen, N. B. (2020). Prevention of internalizing disorders and suicide via adolescent sleep interventions. Current Opinion in Psychology, 34, 37–42. 10.1016/j.copsyc.2019.08.027 31593876

[jcv270082-bib-0009] Blake, M. J. , Blake, L. M. , Schwartz, O. , Raniti, M. , Waloszek, J. M. , Murray, G. , Simmons, J. G. , Landau, E. , Dahl, R. E. , McMakin, D. L. , Dudgeon, P. , Trinder, J. , & Allen, N. B. (2018). Who benefits from adolescent sleep interventions? Moderators of treatment efficacy in a randomized controlled trial of a cognitive‐behavioral and mindfulness‐based group sleep intervention for at‐risk adolescents. Journal of Child Psychology and Psychiatry, 59(6), 637–649. 10.1111/jcpp.12842 29164609

[jcv270082-bib-0010] Blake, M. J. , Trinder, J. A. , & Allen, N. B. (2018a). Mechanisms underlying the association between insomnia, anxiety, and depression in adolescence: Implications for behavioral sleep interventions. Clinical Psychology Review, 63, 25–40. 10.1016/j.cpr.2018.05.006 29879564

[jcv270082-bib-0011] Boland, E. M. , Goldschmied, J. R. , & Gehrman, P. R. (2023). Does insomnia treatment prevent depression? Sleep, 46(6), zsad104. 10.1093/sleep/zsad104 37029781 PMC10262035

[jcv270082-bib-0012] Brown, W. J. , Wilkerson, A. K. , Boyd, S. J. , Dewey, D. , Mesa, F. , & Bunnell, B. E. (2018). A review of sleep disturbance in children and adolescents with anxiety. Journal of Sleep Research, 27(3), e12635. 10.1111/jsr.12635 29193443

[jcv270082-bib-0013] Bürkner, P. C. (2017). Brms: An R package for bayesian multilevel models using stan. Journal of Statistical Software, 80(1), 1–28. 10.18637/jss.v080.i01

[jcv270082-bib-0014] Buysse, D. J. (2014). Sleep health: Can we define it? Does it matter? Sleep, 37(1), 9–17. 10.5665/sleep.3298 24470692 PMC3902880

[jcv270082-bib-0015] Casey, B. j. , Jones, R. M. , & Hare, T. A. (2008). The adolescent brain. Annals of the New York Academy of Sciences, 1124(1), 111–126. 10.1196/annals.1440.010 18400927 PMC2475802

[jcv270082-bib-0016] Castiglione‐Fontanellaz, C. E. G. , Schaufler, S. , Wild, S. , Hamann, C. , Kaess, M. , & Tarokh, L. (2023). Sleep regularity in healthy adolescents: Associations with sleep duration, sleep quality, and mental health. Journal of Sleep Research, 32(4), e13865. 10.1111/jsr.13865 36852716

[jcv270082-bib-0017] Chachos, E. , Shen, L. , Yap, Y. , Maskevich, S. , Stone, J. E. , Wiley, J. F. , & Bei, B. (2023). Vulnerability to sleep‐related affective disturbances? A closer look at dysfunctional beliefs and attitudes about sleep as a moderator of daily sleep‐affect associations in young people. Sleep Health, 9(5), 672–679. 10.1016/j.sleh.2023.07.008 37640630

[jcv270082-bib-0018] Clayborne, Z. M. , Varin, M. , & Colman, I. (2019). Systematic review and meta‐analysis: Adolescent depression and long‐term psychosocial outcomes. Journal of the American Academy of Child & Adolescent Psychiatry, 58(1), 72–79. 10.1016/j.jaac.2018.07.896 30577941

[jcv270082-bib-0019] Cloos, L. , Ceulemans, E. , & Kuppens, P. (2023). Development, validation, and comparison of self‐report measures for positive and negative affect in intensive longitudinal research. Psychological Assessment, 35(3), 189–204. 10.1037/pas0001200 36480406

[jcv270082-bib-0020] Crouse, J. J. , Carpenter, J. S. , Song, Y. J. C. , Hockey, S. J. , Naismith, S. L. , Grunstein, R. R. , Scott, E. M. , Merikangas, K. R. , Scott, J. , & Hickie, I. B. (2021). Circadian rhythm sleep–wake disturbances and depression in young people: Implications for prevention and early intervention. The Lancet Psychiatry, 8(9), 813–823. 10.1016/S2215-0366(21)00034-1 34419186

[jcv270082-bib-0021] Crowley, S. J. , Wolfson, A. R. , Tarokh, L. , & Carskadon, M. A. (2018). An update on adolescent sleep: New evidence informing the perfect storm model. Journal of Adolescence, 67(1), 55–65. 10.1016/j.adolescence.2018.06.001 29908393 PMC6054480

[jcv270082-bib-0022] Drexl K. , Urben S. , Plessen K. J. , Glaus J. (2023). The daily interplay of sleep and affect in subclinical internalizing symptoms in adolescents. 10.17605/OSF.IO/S2JM6

[jcv270082-bib-0023] Drexl, K. , Urben, S. , Plessen, K. J. , & Glaus, J. (2024). Using mobile assessments to characterize mental and physical health behaviors in youth: The mSanté protocol for a pilot observational intensive longitudinal study. OSF. 10.31219/osf.io/e49yc PMC12539651 41118483

[jcv270082-bib-0024] Drexl, K. , Urben, S. , Plessen, K. J. , & Glaus, J. (2025). Using Mobile assessments to characterize mental and physical health behaviors in youth: Protocol for a pilot intensive longitudinal study. JMIR Res Protoc, 14(1), e70990. 10.2196/70990 41118483 PMC12539651

[jcv270082-bib-0025] Dzubur, E. , Ponnada, A. , Nordgren, R. , Yang, C. H. , Intille, S. , Dunton, G. , & Hedeker, D. (2020). MixWILD: A program for examining the effects of variance and slope of time‐varying variables in intensive longitudinal data. Behavior Research Methods, 52(4), 1403–1427. 10.3758/s13428-019-01322-1 31898295 PMC7406537

[jcv270082-bib-0026] Elmer, T. , Ram, N. , Gloster, A. T. , & Bringmann, L. F. (2023). Studying daily social interaction quantity and quality in relation to depression change: A multi‐phase experience sampling study. Personality and Social Psychology Bulletin, 51(7), 01461672231211469. 10.1177/01461672231211469 PMC12130595 38098172

[jcv270082-bib-0027] El‐Sheikh, M. , Gillis, B. T. , Saini, E. K. , Erath, S. A. , & Buckhalt, J. A. (2022). Sleep and disparities in child and adolescent development. Child Dev Perspect, 16(4), 200–207. 10.1111/cdep.12465 36337834 PMC9629655

[jcv270082-bib-0028] Essau, C. A. , Lewinsohn, P. M. , Olaya, B. , & Seeley, J. R. (2014). Anxiety disorders in adolescents and psychosocial outcomes at age 30. Journal of Affective Disorders, 163, 125–132. 10.1016/j.jad.2013.12.033 24456837 PMC4028371

[jcv270082-bib-0029] Faulstich, M. E. , Carey, M. P. , Ruggiero, L. , Enyart, P. , & Gresham, F. (1986). Assessment of depression in childhood and adolescence: An evaluation of the center for epidemiological studies depression scale for children (CES‐DC). American Journal of Psychiatry, 143(8), 1024–1027. 10.1176/ajp.143.8.1024 3728717

[jcv270082-bib-0030] Fuhrer, R. , & Rouillon, F. (1989). La version française de l’échelle CES‐D (Center for Epidemiologic Studies‐Depression Scale). Description et traduction de l’échelle d’autoévaluation. Psychiatry & Psychobiology, 4(3), 163–166. 10.1017/S0767399X00001590

[jcv270082-bib-0031] Fuligni, A. J. , Arruda, E. H. , Krull, J. L. , & Gonzales, N. A. (2018). Adolescent sleep duration, variability, and peak levels of achievement and mental health. Child Development, 89(2), e18–e28. 10.1111/cdev.12729 28129442 PMC5529284

[jcv270082-bib-0032] Fusar‐Poli, P. , Correll, C. U. , Arango, C. , Berk, M. , Patel, V. , & Ioannidis, J. P. A. (2021). Preventive psychiatry: A blueprint for improving the mental health of young people. World Psychiatry, 20(2), 200–221. 10.1002/wps.20869 34002494 PMC8129854

[jcv270082-bib-0033] Galván, A. (2020). The need for sleep in the adolescent brain. Trends in Cognitive Sciences, 24(1), 79–89. 10.1016/j.tics.2019.11.002 31780247

[jcv270082-bib-0034] Gariepy, G. , Danna, S. , Gobiņa, I. , Rasmussen, M. , Gaspar de Matos, M. , Tynjälä, J. , Janssen, I., Ph.D. , Kalman, M., Ph.D. , Villeruša, A. , Husarova, D. , Brooks, F. , Elgar, F. J. , Klavina‐Makrecka, S., M.Sc. , Šmigelskas, K. , Gaspar, T. , & Schnohr, C. (2020). How are adolescents sleeping? Adolescent sleep patterns and sociodemographic differences in 24 European and North American countries. Journal of Adolescent Health, 66(6, Supplement), S81–S88. 10.1016/j.jadohealth.2020.03.013 32446613

[jcv270082-bib-0035] GBD 2019 Mental Disorders Collaborators . (2022). Global, regional, and national burden of 12 mental disorders in 204 countries and territories, 1990–2019: A systematic analysis for the global burden of disease study 2019. The Lancet Psychiatry, 9(2), 137–150. 10.1016/S2215-0366(21)00395-3 35026139 PMC8776563

[jcv270082-bib-0036] Gelman, A. (2006). Prior distributions for variance parameters in hierarchical models (comment on article by browne and draper). Bayesian Anal, 1(3), 515–534. 10.1214/06-BA117A

[jcv270082-bib-0037] Giddens, N. T. , Juneau, P. , Manza, P. , Wiers, C. E. , & Volkow, N. D. (2022). Disparities in sleep duration among American children: Effects of race and ethnicity, income, age, and sex. Proceedings of the National Academy of Sciences, 119(30), e2120009119. 10.1073/pnas.2120009119 PMC9335336 35858412

[jcv270082-bib-0038] Gradisar, M. , Kahn, M. , Micic, G. , Short, M. , Reynolds, C. , Orchard, F. , Bauducco, S. , Bartel, K. , & Richardson, C. (2022). Sleep’s role in the development and resolution of adolescent depression. Nature Reviews Psychology, 1(9), 512–523. 10.1038/s44159-022-00074-8 PMC9208261 35754789

[jcv270082-bib-0039] Hallensleben, N. , Kraiss, J. , Glaesmer, H. , Forkmann, T. , & Spangenberg, L. (2024). Examining heterogeneity in the affect‐regulating function of suicidal ideation: Person‐specific analyses in male inpatients with depression. Suicide and Life‐Threatening Behavior, 54(6), 1123–1132. 10.1111/sltb.13117 39023190 PMC11629603

[jcv270082-bib-0040] Hamaker, E. L. , & Grasman, R. P. P. P. (2015). To center or not to center? Investigating inertia with a multilevel autoregressive model. Frontiers in Psychology, 5, 1492. 10.3389/fpsyg.2014.01492 25688215 PMC4310502

[jcv270082-bib-0041] Harris, P. A. , Taylor, R. , Minor, B. L. , Elliott, V. , Fernandez, M. , O'Neal, L. , McLeod, L. , Delacqua, G. , Delacqua, F. , Kirby, J. , & Duda, S. N. (2019). The REDCap consortium: Building an international community of software platform partners. Journal of Biomedical Informatics, 95, 103208. 10.1016/j.jbi.2019.103208 31078660 PMC7254481

[jcv270082-bib-0042] Harris, P. A. , Taylor, R. , Thielke, R. , Payne, J. , Gonzalez, N. , & Conde, J. G. (2009). Research electronic data capture (REDCap)—A metadata‐driven methodology and workflow process for providing translational research informatics support. Journal of Biomedical Informatics, 42(2), 377–381. 10.1016/j.jbi.2008.08.010 18929686 PMC2700030

[jcv270082-bib-0043] Haslbeck, J. M. B. , & Ryan, O. (2022). Recovering within‐person dynamics from psychological time series. Multivariate Behavioral Research, 57(5), 735–766. 10.1080/00273171.2021.1896353 34154483

[jcv270082-bib-0044] Hickman, R. , D’Oliveira, T. C. , Davies, A. , & Shergill, S. (2024). Monitoring daily sleep, mood, and affect using digital technologies and wearables: A systematic review. Sensors, 24(14), 4701. 10.3390/s24144701 39066098 PMC11280943

[jcv270082-bib-0045] Kelly, R. J. , Zeringue, M. M. , & El‐Sheikh, M. (2022). Adolescents’ sleep and adjustment: Reciprocal effects. Child Development, 93(2), 540–555. 10.1111/cdev.13703 34757645 PMC8930734

[jcv270082-bib-0046] Könen, T. , Dirk, J. , Leonhardt, A. , & Schmiedek, F. (2016). The interplay between sleep behavior and affect in elementary school children’s daily life. Journal of Experimental Child Psychology, 150, 1–15. 10.1016/j.jecp.2016.04.003 27236036

[jcv270082-bib-0047] Kraiss, J. , Glaesmer, H. , Forkmann, T. , Spangenberg, L. , Hallensleben, N. , Schreiber, D. , & Höller, I. (2024). Beyond one‐size‐fits‐all suicide prediction: Studying idiographic associations of risk factors for suicide in a psychiatric sample using ecological momentary assessment. Journal of Psychiatric Research, 178, 130–138. 10.1016/j.jpsychires.2024.07.050 39141992

[jcv270082-bib-0048] Krokstad, M. A. , Sund, E. , Rangul, V. , Bauman, A. , Olsson, C. , & Bjerkeset, O. (2024). Secular trends in risk factors for adolescent anxiety and depression symptoms: The Young‐HUNT studies 1995–2019, Norway. European Child & Adolescent Psychiatry, 33(11), 3819–3827. 10.1007/s00787-024-02373-2 38578474 PMC11588762

[jcv270082-bib-0049] Larsen, R. J. , & Diener, E. (1992). Promises and problems with the circumplex model of emotion. Emotion, 25–59.

[jcv270082-bib-0050] Lee, M. D. , & Wagenmakers, E. J. (2014). Bayesian cognitive modeling: A practical course. Cambridge University Press.

[jcv270082-bib-0051] Lemke, T. , Hökby, S. , Wasserman, D. , Carli, V. , & Hadlaczky, G. (2023). Associations between sleep habits, quality, chronotype and depression in a large cross‐sectional sample of Swedish adolescents. PLoS One, 18(11), e0293580. 10.1371/journal.pone.0293580 37917651 PMC10621812

[jcv270082-bib-0052] Lok, R. , Weed, L. , Winer, J. , & Zeitzer, J. M. (2024). Perils of the nighttime: Impact of behavioral timing and preference on mental health in 73,888 community‐dwelling adults. Psychiatry Research, 337, 115956. 10.1016/j.psychres.2024.115956 38763081

[jcv270082-bib-0053] Lollies, F. , Schnatschmidt, M. , Bihlmeier, I. , Genuneit, J. , In‐Albnon, T. , Holtmann, M. , Legenbauer, T. , & Schlarb, A. A. (2022). Associations of sleep and emotion regulation processes in childhood and adolescence—a systematic review, report of methodological challenges and future directions. Sleep Sci, 15(4), 490–514. 10.5935/1984-0063.20220082 36419813 PMC9670771

[jcv270082-bib-0054] Lovato, N. , & Gradisar, M. (2014). A meta‐analysis and model of the relationship between sleep and depression in adolescents: Recommendations for future research and clinical practice. Sleep Medicine Reviews, 18(6), 521–529. 10.1016/j.smrv.2014.03.006 24857255

[jcv270082-bib-0055] Lunsford‐Avery, J. R. , Wang, K. W. , Kollins, S. H. , Chung, R. J. , Keller, C. , & Engelhard, M. M. (2022). Regularity and timing of sleep patterns and behavioral health among adolescents. Journal of Developmental and Behavioral Pediatrics, 43(4), 188–196. 10.1097/DBP.0000000000001013 34698705 PMC9035469

[jcv270082-bib-0056] Maskevich, S. , Shen, L. , Drummond, S. P. A. , & Bei, B. (2021). What time do you plan to sleep tonight? An intense longitudinal study of adolescent daily sleep self‐regulation via planning and its associations with sleep opportunity. Journal of Child Psychology and Psychiatry, 22(8), 22–911. 10.1111/jcpp.13540 34811748

[jcv270082-bib-0057] Mathew, G. M. , Li, X. , Hale, L. , & Chang, A. M. (2019). Sleep duration and social jetlag are independently associated with anxious symptoms in adolescents. Chronobiology International, 36(4), 461–469. 10.1080/07420528.2018.1509079 30786775 PMC6397070

[jcv270082-bib-0058] Messman, B. A. , Slavish, D. C. , Briggs, M. , Ruggero, C. J. , Luft, B. J. , & Kotov, R. (2023). Daily sleep‐stress reactivity and functional impairment in world trade center responders. Ann Behav Med Publ Soc Behav Med., 57(7), 582–592. 10.1093/abm/kaad005 37078921

[jcv270082-bib-0059] Messman, B. A. , Wiley, J. F. , Feldman, E. , Dietch, J. R. , Taylor, D. J. , & Slavish, D. C. (2024). Irregular sleep is linked to poorer mental health: A pooled analysis of eight studies. Sleep Health, 10(4), 493–499. 10.1016/j.sleh.2024.03.004 38704353 PMC12036700

[jcv270082-bib-0060] Migueles, J. H. , Rowlands, A. V. , Huber, F. , Sabia, S. , & Van Hees, V. T. (2019). GGIR: A research community–driven open source R package for generating physical activity and sleep outcomes from multi‐day raw accelerometer data. J Meas Phys Behav, 2(3), 188–196. 10.1123/jmpb.2018-0063

[jcv270082-bib-0061] Monterastelli, A. J. , Adams, J. , Eastman, C. I. , & Crowley, S. J. (2024). The forbidden zone for sleep is more robust in adolescents compared to adults. Front Sleep, 2, 1304647. 10.3389/frsle.2023.1304647 39917052 PMC11801367

[jcv270082-bib-0062] Murray, E. J. , & Kunicki, Z. (2022). As the wheel turns: Causal inference for feedback loops and bidirectional effects. OSF. 10.31219/osf.io/9em5q

[jcv270082-bib-0063] Neubauer, A. B. , & Schmiedek, F. (2020). Studying within‐person variation and within‐person couplings in intensive longitudinal data: Lessons learned and to be learned. Gerontology, 66(4), 332–339. 10.1159/000507993 32526756

[jcv270082-bib-0064] Neubauer, A. B. , Voelkle, M. C. , Voss, A. , & Mertens, U. K. (2020). Estimating reliability of within‐person couplings in a multilevel framework. Journal of Personality Assessment, 102(1), 10–21. 10.1080/00223891.2018.1521418 30633577

[jcv270082-bib-0065] Nutt, D. , Wilson, S. , & Paterson, L. (2008). Sleep disorders as core symptoms of depression. Dialogues in Clinical Neuroscience, 10(3), 329–336. 10.31887/DCNS.2008.10.3/dnutt 18979946 PMC3181883

[jcv270082-bib-0066] Palmer, C. A. , Bower, J. L. , Cho, K. W. , Clementi, M. A. , Lau, S. , Oosterhoff, B. , & Alfano, C. A. (2024). Sleep loss and emotion: A systematic review and meta‐analysis of over 50 years of experimental research. Psychological Bulletin, 150(4), 440–463. 10.1037/bul0000410 38127505

[jcv270082-bib-0067] Phillips, A. J. K. , Clerx, W. M. , O’Brien, C. S. , Sano, A. , Barger, L. K. , Picard, R. W. , Lockley, S. W. , Klerman, E. B. , & Czeisler, C. A. (2017). Irregular sleep/wake patterns are associated with poorer academic performance and delayed circadian and sleep/wake timing. Scientific Reports, 7(1), 3216. 10.1038/s41598-017-03171-4 28607474 PMC5468315

[jcv270082-bib-0068] Putilov, A. A. (2023). Weekend sleep after early and later school start times confirmed a model‐predicted failure to catch up sleep missed on weekdays. Sleep and Breathing, 27(2), 709–719. 10.1007/s11325-022-02648-5 35657472 PMC9164574

[jcv270082-bib-0069] R Core Team . (2023). R: A language and environment for statistical computing. Retrieved from https://www.R‐project.org/

[jcv270082-bib-0070] Rice, F. , Eyre, O. , Riglin, L. , & Potter, R. (2017). Adolescent depression and the treatment gap. The Lancet Psychiatry, 4(2), 86–87. 10.1016/S2215-0366(17)30004-4 28087200

[jcv270082-bib-0071] Riemann, D. , Dressle, R. J. , Benz, F. , Spiegelhalder, K. , Johann, A. F. , Nissen, C. , Hertenstein, E. , Baglioni, C. , Palagini, L. , Krone, L. , Perlis, M. L. , Domschke, K. , Berger, M. , & Feige, B. (2024). Chronic insomnia, REM sleep instability and emotional dysregulation: A pathway to anxiety and depression? Journal of Sleep Research, 34(2), e14252. 10.1111/jsr.14252 38811745 PMC11911052

[jcv270082-bib-0072] Roenneberg, T. , Kuehnle, T. , Pramstaller, P. P. , Ricken, J. , Havel, M. , Guth, A. , & Merrow, M. (2004). A marker for the end of adolescence. Current Biology, 14(24), R1038–R1039. 10.1016/j.cub.2004.11.039 15620633

[jcv270082-bib-0073] Roenneberg, T. , Pilz, L. K. , Zerbini, G. , & Winnebeck, E. C. (2019). Chronotype and social Jetlag: A (Self‐) critical review. Biology, 8(3), 54. 10.3390/biology8030054 31336976 PMC6784249

[jcv270082-bib-0074] Savord, A. , McNeish, D. , Iida, M. , Quiroz, S. , & Ha, T. (2023). Fitting the longitudinal actor‐partner interdependence model as a dynamic structural equation model in mplus. Struct Equ Model Multidiscip J, 30(2), 296–314. 10.1080/10705511.2022.2065279

[jcv270082-bib-0075] Shen, L. , Wiley, J. F. , & Bei, B. (2022). Sleep and affect in adolescents: Bidirectional daily associations over 28‐day ecological momentary assessment. Journal of Sleep Research, 31(2), e13491. 10.1111/jsr.13491 34585468

[jcv270082-bib-0076] Skeldon, A. C. , Derks, G. , & Dijk, D. J. (2016). Modelling changes in sleep timing and duration across the lifespan: Changes in circadian rhythmicity or sleep homeostasis? Sleep Medicine Reviews, 28, 96–107. 10.1016/j.smrv.2015.05.011 26545247

[jcv270082-bib-0077] Skorucak, J. , Weber, N. , Carskadon, M. A. , Reynolds, C. , Coussens, S. , Achermann, P. , & Short, M. A. (2021). Homeostatic response to sleep restriction in adolescents. Sleep, 44(9), zsab106. 10.1093/sleep/zsab106 33893807

[jcv270082-bib-0078] Spiegelberger, C. , Edwards, C. , Montouri, J. , & Lushene, R. (1973). Preliminary test manual for the state–trait anxiety inventory for childre. Consulting Psychologists Press.

[jcv270082-bib-0079] Tamura, N. , & Okamura, K. (2024). Longitudinal course and outcome of social jetlag in adolescents: A 1‐year follow‐up study of the adolescent sleep health epidemiological cohorts. Journal of Sleep Research, 33(3), e14042. 10.1111/jsr.14042 37697814

[jcv270082-bib-0080] Textor, J. , & Liskiewicz, M. (2012). Adjustment criteria in causal diagrams: An algorithmic perspective. arXiv. 10.48550/arXiv.1202.3764

[jcv270082-bib-0081] Tomaso, C. C. , Johnson, A. B. , & Nelson, T. D. (2021). The effect of sleep deprivation and restriction on mood, emotion, and emotion regulation: Three meta‐analyses in one. Sleep, 44(6), zsaa289. 10.1093/sleep/zsaa289 33367799 PMC8193556

[jcv270082-bib-0082] Turgeon, L. , & Chartrand, É (2003). Psychometric properties of the French Canadian version of the state‐trait anxiety inventory for children. Educational and Psychological Measurement, 63(1), 174–185. 10.1177/0013164402239324

[jcv270082-bib-0083] Usami, S. , Murayama, K. , & Hamaker, E. L. (2019). A unified framework of longitudinal models to examine reciprocal relations. Psychological Methods, 24(5), 637–657. 10.1037/met0000210 30998041

[jcv270082-bib-0084] Van Hees, V. T. (2024). GGIR 3.1‐3 release notes. https://github.com/wadpac/GGIR/releases/tag/3.1‐3. Accessed October 7, 2024.

[jcv270082-bib-0085] van Hees, V. T. , Fang, Z. , Zhao, J. H. , Heywood, J. , Mirkes, E. , Sabia, S. , & Migueles, J. M. (2022). GGIR: Raw accelerometer data analysis. R‐package version 2.8‐2. 10.5281/zenodo.1051064

[jcv270082-bib-0086] van Hees, V. T. , Sabia, S. , Anderson, K. N. , Denton, S. J. , Oliver, J. , Catt, M. , Abell, J. G. , Kivimäki, M. , Trenell, M. I. , & Singh‐Manoux, A. (2015). A novel, open access method to assess sleep duration using a wrist‐worn accelerometer. PLoS One, 10(11), e0142533. 10.1371/journal.pone.0142533 26569414 PMC4646630

[jcv270082-bib-0087] Van Hees, V. T. , Sabia, S. , Jones, S. E. , Wood, A. R. , Anderson, K. N. , Kivimäki, M. , Frayling, T. M. , Pack, A. I. , Bucan, M. , Trenell, M. I. , Mazzotti, D. R. , Gehrman, P. R. , Singh‐Manoux, B. A. , & Weedon, M. N. (2018). Estimating sleep parameters using an accelerometer without sleep diary. Scientific Reports, 8(1), 12975. 10.1038/s41598-018-31266-z 30154500 PMC6113241

[jcv270082-bib-0088] Vehtari, A. , Gelman, A. , & Gabry, J. (2017). Practical Bayesian model evaluation using leave‐one‐out cross‐validation and WAIC. Statistics and Computing, 27(5), 1413–1432. 10.1007/s11222-016-9696-4

[jcv270082-bib-0089] Vehtari, A. , Simpson, D. , Gelman, A. , Yao, Y. , & Gabry, J. (2022). Pareto smoothed importance sampling. arXiv. 10.48550/arXiv.1507.02646

[jcv270082-bib-0090] Vert, A. , Weber, K. S. , Thai, V. , Turner, E. , Beyer, K. B. , Cornish, B. F. , Godkin, F. E. , Wong, C. , McIlroy, W. E. , & Van Ooteghem, K. (2022). Detecting accelerometer non‐wear periods using change in acceleration combined with rate‐of‐change in temperature. BMC Medical Research Methodology, 22(1), 147. 10.1186/s12874-022-01633-6 35596151 PMC9123693

[jcv270082-bib-0091] Vidal Bustamante, C. M. , Rodman, A. M. , Dennison, M. J. , Flournoy, J. C. , Mair, P. , & McLaughlin, K. A. (2020). Within‐person fluctuations in stressful life events, sleep, and anxiety and depression symptoms during adolescence: A multiwave prospective study. Journal of Child Psychology and Psychiatry, 61(10), 1116–1125. 10.1111/jcpp.13234 32185808 PMC7494581

[jcv270082-bib-0092] Vidal‐Ribas, P. (2025). Editorial: A granular approach to internalizing disorders in adolescents: Examining developmental pathways and environmental influences to identify high‐risk youth. JCPP Adv, 5(1), e70000. n/a(n/a). 10.1002/jcv2.70000 40059995 PMC11889641

[jcv270082-bib-0093] Yan, J. , Xie, M. , Zhao, Z. , Bae, J. , Cham, H. , El‐Sheikh, M. , & Yip, T. (2024). Sleep difficulties and adolescent internalising symptoms: The moderating role of sleep regularity. Journal of Sleep Research, 34(5), e14481. 10.1111/jsr.14481 PMC12353120 40012407

