# Supplementary material for: Toward an idiographic understanding of the role of sleep‐mood dynamics in adolescents' internalizing symptoms
Source: JCPP Adv. 2025 Dec 17;6(3):e70082. doi: 10.1002/jcv2.70082 (PMC13339598; doi:10.1002/jcv2.70082)
Supplement: Supplementary file 1 [file JCV2-6-e70082-s001.docx]

**Towards an idiographic understanding of the role of sleep-mood dynamics in adolescents’ internalizing symptoms**

**Supporting Information**

Contents

[Appendix S1. Deviations from the registered analysis plan	4](#_Toc213284851)

[Appendix S2. Data availability	6](#_Toc213284852)

[Figure S1. Seasonal distribution of assessment waves	6](#_Toc213284853)

[Combinations of missingness	6](#_Toc213284854)

[Figure S2.1. Missingness on the momentary level for sad mood and depression	7](#_Toc213284855)

[Figure S2.2. Missingness on daily level for sad mood and depression	7](#_Toc213284856)

[Figure S2.3. Missingness on person level for sad mood and depression	8](#_Toc213284857)

[Figure S2.4. Missingness on the momentary level for anxious mood and anxiety symptoms	8](#_Toc213284858)

[Figure S2.5. Missingness on daily level for anxious mood and anxiety symptoms	9](#_Toc213284859)

[Figure S2.6. Missingness on person level for anxious mood and anxiety symptoms	9](#_Toc213284860)

[Appendix S3. Model equations and priors	9](#_Toc213284861)

[Appendix S4. Detailed descriptive results	12](#_Toc213284862)

[Figure S3. Availability of EMA and sleep recordings across participants	12](#_Toc213284863)

[Figure S4. Bivariate associations among variables on the daily level	13](#_Toc213284864)

[Figure S5. Bivariate associations among variables on the person level	14](#_Toc213284865)

[Appendix S5. Detailed results of the primary analysis	14](#_Toc213284866)

[Within-person models (Step-1)	14](#_Toc213284867)

[Table S1.1. Sleep duration predicting next-day mood.	14](#_Toc213284868)

[Table S1.2. Sleep midpoint predicting next-day mood.	15](#_Toc213284869)

[Table S1.3. Sleep regularity predicting next-day mood.	16](#_Toc213284870)

[Between-person models (Step 2)	17](#_Toc213284871)

[Table S2.1. Sleep duration couplings predicting internalizing symptoms.	17](#_Toc213284872)

[Table S2.2. Sleep midpoint couplings predicting internalizing symptoms.	17](#_Toc213284873)

[Table S2.3. Sleep regularity couplings predicting internalizing symptoms.	18](#_Toc213284874)

[Collinearity between predictors of symptoms	18](#_Toc213284875)

[Table S3. Matrix of zero-order correlations and partial-correlations	18](#_Toc213284876)

[Appendix S6. Results based on the registered dataset	19](#_Toc213284877)

[Table S4. Variation in random slopes	19](#_Toc213284878)

[Table S5. Between-person associations of sleep-mood couplings and internalizing symptoms	19](#_Toc213284879)

[Appendix S7. Sensitivity Analyses	20](#_Toc213284880)

[Figure S6. Prior sensitivity analysis for variation in random slopes	20](#_Toc213284881)

[Figure S7. Prior sensitivity analysis for prediction of internalizing symptoms	21](#_Toc213284882)

[Figure S8. Influential observations on the daily level	22](#_Toc213284883)

[Figure S9. Influential observations on the person-level	23](#_Toc213284884)

[Table S6. Between-person models without influential observations	24](#_Toc213284885)

[Restrictive inclusion	25](#_Toc213284886)

[Explicit person-mean-centering of the Sleep Regularity Index	26](#_Toc213284887)

[Table S7. Results of person-mean-centered sleep regularity.	26](#_Toc213284888)

[Alternative symptom outcomes	26](#_Toc213284889)

[Table S8. Average weekly symptoms of depression and anxiety	26](#_Toc213284890)

[Table S9. Internalizing subscale of the Youth Self-Report	27](#_Toc213284891)

[Appendix S8. Registered exploratory analyses	27](#_Toc213284892)

[Within-Person Coupling Reliability	27](#_Toc213284893)

[Table S10.1. Incremental Within-person Coupling Reliability for sleep duration and sad mood	27](#_Toc213284894)

[Table S10.2. Incremental Within-person Coupling Reliability for sleep midpoint and sad mood	29](#_Toc213284895)

[Table S10.3. Incremental Within-person Coupling Reliability for sleep regularity and sad mood	29](#_Toc213284896)

[Table S10.4. Incremental Within-person Coupling Reliability for sleep duration and anxious mood	30](#_Toc213284897)

[Table S10.5. Incremental Within-person Coupling Reliability for sleep midpoint and anxious	31](#_Toc213284898)

[Table S10.6. Incremental Within-person Coupling Reliability for sleep midpoint and anxious	31](#_Toc213284899)

[Modeling residual variance and multivariate outcomes	32](#_Toc213284900)

[Interactions with within-person couplings	33](#_Toc213284901)

[Table S11.1. Model comparison for couplings of sleep duration and sad mood	33](#_Toc213284902)

[Table S11.2. Model comparison for couplings of sleep midpoint and sad mood	33](#_Toc213284903)

[Table S11.3. Model comparison for couplings of sleep regularity and sad mood	34](#_Toc213284904)

[Table S11.4. Model comparison for couplings of sleep duration and anxious mood	34](#_Toc213284905)

[Table S11.5. Model comparison for couplings of sleep midpoint and anxious mood	35](#_Toc213284906)

[Table S11.6. Model comparison for couplings of sleep regularity and anxious mood	35](#_Toc213284907)

[Nonlinear couplings of sleep patterns and daytime mood	35](#_Toc213284908)

[Table S12.1. Model comparison for couplings of sleep duration and sad mood	36](#_Toc213284909)

[Table S12.2. Model comparison for couplings of sleep midpoint and sad mood	36](#_Toc213284910)

[Table S12.3. Model comparison for couplings of sleep regularity and sad mood	36](#_Toc213284911)

[Table S12.4. Model comparison for couplings of sleep duration and anxious mood	36](#_Toc213284912)

[Table S12.5. Model comparison for couplings of sleep duration and anxious mood	37](#_Toc213284913)

[Table S12.6. Model comparison for couplings of sleep regularity and anxious mood	37](#_Toc213284914)

[Pubertal maturation	37](#_Toc213284915)

[Table S13. Interaction of pubertal development with sleep patterns	38](#_Toc213284916)

[Table S14. Interaction of pubertal development with sleep-mood couplings predicting internalizing symptoms	38](#_Toc213284917)

[Alternative sleep variables	39](#_Toc213284918)

[Table S15. Variation in random slopes for alternative sleep indicators	39](#_Toc213284919)

[Table S16. Associations of sleep-mood couplings and internalizing symptoms	40](#_Toc213284920)

[Figure S10. Correlations among daily mood and sleep variables	41](#_Toc213284921)

[Figure S11. Partial correlations among daily mood and sleep variables	42](#_Toc213284922)

[Circadian preference	43](#_Toc213284923)

[Table S17. Interaction of circadian preference with sleep patterns	43](#_Toc213284924)

[Table S18. Interaction of circadian preference with sleep-mood couplings predicting internalizing symptoms	44](#_Toc213284925)

[References	45](#_Toc213284926)

# Appendix S1. Deviations from the registered analysis plan

The hypotheses and analysis plan for the present study were registered at an early stage of data collection. In this section, we transparently document deviations from the registered analysis plan and discuss analytical decisions that were not explicitly addressed therein.

Regarding sample and protocol adjustments, the original registration outlined the inclusion of a preliminary subset of participants and assessment waves completed within the first year of a two-year data collection period. However, data collection was ongoing concurrently across multiple schools, resulting in overlapping schedules and substantial logistical demands on the research team. To leverage the full extent of collected data, we opted to extend the analytic dataset to include all adolescents recruited throughout the entire project duration. This expansion aligns with the principles of Bayesian updating, facilitating more robust and decisive evidence by incorporating all available data points.

Additionally, the initially registered analytical approach based on the joint multilevel modeling proposed by Parker et al.(2021) encountered convergence issues. Although the registered analysis plan provided a fallback two-step approach—specifically, a weighted regression using uncertainty estimates from random slopes as weights, aligning with individual patient metaanalysis—we ultimately implemented an alternative two-step modelization inspired by a method specifically developed for intensive longitudinal data (Dzubur et al., 2020). This adaptation involved translating the frequentist methodology into a Bayesian estimation framework, utilizing empirical posterior distributions of model parameters rather than theoretical distributions based on point estimates and standard errors. Consequently, our final analyses involved extensive computational demand, totaling approximately 7,000,000 post-warmup draws per Step-2 model. For transparency, we include results from the originally planned weighted regression analyses based on the registered dataset in this appendix.

Considering the interpretability of Step-2 models, we chose to report in the main text only those analyses for which heterogeneity in within-person couplings (WPCs) achieved a Bayes Factor of 3 or higher. However, for full transparency, all six Step-2 models are comprehensively presented within this appendix.

The transition to the modified two-step analytical approach allowed for refinement of participant inclusion criteria. Specifically, the initial within-person models (Step-1) did not require participants to have available internalizing symptom scores, thereby enabling an inclusive analysis of all available data related to sleep-mood coupling. This inclusive strategy was intentionally adopted to achieve decisive evidence. Consequently, fewer participants were ultimately included in Step-2 analyses due to missing symptom data. Nevertheless, the information from these participants indirectly influenced the predictors via partial pooling of individual WPC estimates. Although we acknowledge that partial pooling beneficially guards against overfitting, it also inherently reduces observed heterogeneity in WPC estimates.

In reporting within-person model results, we included one-sided Bayes Factors for fixed within-person effects, an approach not explicitly mentioned in the registration. This additional quantification permits clearer differentiation between heterogeneity of individual WPCs spreading around a directional general effect versus a null effect.

Furthermore, our interpretation of WPCs extended beyond magnitude and direction, also considering the the range of predicted values on the absolute scale of the Ecological Momentary Assessment (EMA) items. This comprehensive interpretation became necessary as daily mood responses predominantly occurred within positive ranges of bipolar scales (e.g., happy and calm mood). This nuanced interpretation aims to align with the measures despite the registration was primarily focused on negative mood states (e.g., sad and anxious mood).

Regarding sensitivity analyses on prior specifications, our registration initially anticipated assessing sensitivity only to wider prior distributions for key parameters—specifically, the estimated standard deviation of random slopes and regression parameters predicting between-person symptom differences. In the current analyses, we additionally explored narrower prior distributions and varied the location of priors for random slope standard deviations. All results from these sensitivity checks are presented comprehensively in this appendix.

Most exploratory analyses proceeded as initially registered and are reported herein. However, several advanced modeling strategies described in the registration could not be conducted due to persistent convergence issues even within the modified two-step framework. These unresolved approaches include: (1) modeling individual residual variance using Mixed-Effects Location-Scale Models; (2) joint modeling of sad and anxious mood outcomes via Multivariate Mixed-Effects Location Scale Models; and (3) implementation of three-level multilevel models incorporating the assessment wave as an intermediate hierarchical level. Additional exploratory analyses were conducted, including alternative symptom outcomes: specifically, examining whether WPCs predicted average depression and anxiety symptoms across all weekly assessments allowing maximum inclusion rather than symptoms from only the final monitoring week were we could not compensate for missingness. We further explored predicting the Internalizing subscale of the Youth Self-Report (YSR; Achenbach & Rescorla, 2001) follow-up assessment, adjusting for baseline levels as a covariate.

# Appendix S2. Data availability

## Figure S1. Seasonal distribution of assessment waves

*Note*. The duration of each assessment wave was 21 days. Individual horizontal offsets result from postponed launches when individual students could not attend the respective collective launch with their peers. Missing bars indicate dropout.

## Combinations of missingness

The following upset-plots show combinations of our registered missingness and valdity criteria per outcome (i.e., sad and anxious mood) and per level of aggregation (i.e., momentary, daily, and person-level).

### Figure S2.1. Missingness on the momentary level for sad mood and depression

### Figure S2.2. Missingness on daily level for sad mood and depression

### Figure S2.3. Missingness on person level for sad mood and depression

### Figure S2.4. Missingness on the momentary level for anxious mood and anxiety symptoms

### Figure S2.5. Missingness on daily level for anxious mood and anxiety symptoms

### Figure S2.6. Missingness on person level for anxious mood and anxiety symptoms

# Appendix S3. Model equations and priors

To test the Hypotheses 1a-c and 2a-c, we employed 6 separate Bayesian multilevel models to quantify the variance in the lagged within-person prediction of daily levels of sad or anxious mood by sleep patterns of previous nights.

𝐷𝑎𝑖𝑙𝑦 𝑚𝑜𝑜𝑑:

We specified informative priors based on the published model parameters of the supplemental tables provided by Shen and colleagues (2022) where applicable.

Intercept of mood ratings: 𝛽0(3,2);{1 ≤0≤ 7}

Covariates:

- 𝛽_1_(0.04,0.3)
- 𝛽_2_𝑔𝑒𝑛𝑑𝑒𝑟𝑗(−0.1,0.5)
- 𝛽_3_(0.05,0.5)
- 𝛽_4_𝑤𝑎𝑣𝑒𝑖𝑗(0,0.5)

Fixed effects:

- 𝛽_5__−1,_(0.15,0.3)
- 𝛽_6__._𝑠𝑙𝑒𝑒𝑝𝑗(0.15,1)
- 𝛽_7_𝑠𝑙𝑒𝑒𝑝𝑖𝑗(0.0,0.5)

Random effects:

- Correlation matrix: 𝛺 𝐿𝐾𝐽𝑐𝑜𝑟𝑟( = 2)
- 𝜎𝜖 𝐶𝑎𝑢𝑐*hy*(0.2,0.3);{≥0}.

In Step-2 we performed Bayesian multiple regressions to test Hypotheses 3a-c and 4a-c, regarding the prediction of retrospective depressive and anxious symptoms by within-person couplings obtained from Step-1.

𝑆𝑦𝑚𝑝𝑡𝑜𝑚 𝑠𝑒𝑣𝑒𝑟𝑖𝑡𝑦: 𝑤_𝑗,𝑢1𝑗_𝑦_2_𝑗 = 𝛾_0_ + 𝛾_1_𝑦𝑏𝑎𝑠𝑒𝑙𝑖𝑛𝑒_,_𝑗 + 𝛾_2_𝑢_0_𝑗 + 𝛾_3_𝑢_1_𝑗 + 𝑢_2_𝑗

Where the weight 𝑤_𝑗,𝑢1𝑗_ is the inverse uncertainty in the within-person coupling 𝑢_1𝑗_. Weights were extracted as the mean absolute difference across all Markov-Chain-Monte-Carlo (MCMC) draws for each individual random-slope parameter.

Priors were based on previously published descriptives in healthy adolescent publications:

For depressive symptoms (CES-DC):

- 𝛾_0_(70,20);{20 ≤_0_ ≤ 120}; derived and rescaled from (Rieck et al., 2013)
- 𝜎_𝑢2_ 𝐶𝑎𝑢𝑐(0,10); 𝐼{≥ 0}
- 𝛾_1_(1,5)
- 𝛾_2_(0,10)
- 𝛾_3_(0,10)

For anxiety symptoms (STAIC):

- 𝛾

- 𝜎_𝑢2_ ~ 𝐶𝑎𝑢𝑐*hy* (0,5);{2>=0}
- 𝛾_1_(1,3)
- 𝛾_2_(0,5)
- 𝛾_3_(0,5)

# Appendix S4. Detailed descriptive results

## Figure S3. Availability of EMA and sleep recordings across participants

## Figure S4. Bivariate associations among variables on the daily level

## Figure S5. Bivariate associations among variables on the person level

# Appendix S5. Detailed results of the primary analysis

## Within-person models (Step-1)

### Table S1.1. Sleep duration predicting next-day mood.

**Sad mood	Anxious mood**

*Predictors	Estimates	CI (95%)	Estimates	CI (95%)*

| Intercept | 3.34 | 0.94 – 5.80 | 3.83 | 1.08 – 6.73 | |
| --- | --- | --- | --- | --- | --- |
| Age | 0.04 | -0.07 – 0.16 | -0.03 | -0.17 – 0.10 | |
| Gender: Female | 0.17 | -0.06 – 0.39 | 0.34 | 0.09 – 0.61 | |
| Day of week: Weekend | -0.13 | -0.18 – -0.07 | -0.06 | -0.12 – 0.01 | |
| Wave: Wave 2 | -0.06 | -0.12 – -0.01 | -0.04 | -0.10 – 0.03 | |
| Wave: Wave 3 | -0.09 | -0.15 – -0.02 | -0.01 | -0.09 – 0.06 | |
| Previous day mood | 0.28 | 0.25 – 0.31 | 0.33 | 0.30 – 0.36 | |
| Average Sleep Duration | -0.22 | -0.39 – -0.05 | -0.18 | -0.38 – 0.02 | |
| Sleep Duration (person mean centered) | -0.00 | -0.02 – 0.02 | -0.03 | -0.05 – -0.01 | |
| **Random Effects** σ^2^ | 1.00 |  | 1.00 |  | |
| τ00 | 0.31 |  | 0.40 |  | |
| τ11 | <0.01 |  | < 0.01 |  | |
| ICC | 0.24 |  | 0.29 |  | |
| N | 102 |  | 97 |  | |
| Observations 4087 | | | 4004 | |  |
| Marginal R^2^ / Conditional R^2^ 0.166 / 0.538 | | | 0.202 / 0.590 | |  |
| ICC: Intraclass Correlation Coefficient | | |  | |  |

### Table S1.2. Sleep midpoint predicting next-day mood.

**Sad mood	Anxious mood**

*Predictors	Estimates	CI (95%)	Estimates	CI (95%)*

| Intercept | | -6.75 | -12.28 – -1.27 | -1.34 | | -7.51 – 5.03 | |
| --- | --- | --- | --- | --- | --- | --- | --- |
| Age | | 0.05 | -0.07 – 0.16 | -0.02 | | -0.15 – 0.12 | |
| Gender: Female | | 0.18 | -0.04 – 0.42 | 0.35 | | 0.09 – 0.61 | |
| Day of week: Weekend | | -0.15 | -0.21 – -0.08 | -0.09 | | -0.17 – -0.02 | |
| Wave: Wave 2 | | -0.06 | -0.11 – -0.00 | -0.03 | | -0.10 – 0.03 | |
| Wave: Wave3 | | -0.08 | -0.14 – -0.02 | -0.01 | | -0.08 – 0.06 | |
| Previous day mood | | 0.28 | 0.25 – 0.31 | 0.13 | | -0.09 – 0.34 | |
| Average Sleep Midpoint | | 0.30 | 0.10 – 0.49 | 0.33 | | 0.30 – 0.36 | |
| Sleep Midpoint (person mean centered) | | 0.01 | -0.02 – 0.04 | 0.00 | | -0.03 – 0.04 | |
| **Random Effects** σ^2^ | | 1.00 |  | 1.00 | |  | |
| τ00 | | 0.31 _study_id_ | | | 0.42 _study_id_ | | |
| τ11 | | 0.00 _study_id.smid.pc_ | | | 0.00 _study_id.smid.pc_ | | |
| ICC | | 0.24 | | | 0.29 | | |
| N | | 102 _study_id_ | | | 97 _study_id_ | | |
| Observations | 4087 | | | 4004 | | |  |
| Marginal R^2^ / Conditional R^2^  ICC: Intraclass Correlation Coefficient | 0.171 / 0.539 | | | 0.191 / 0.589 | | |  |

### Table S1.3. Sleep regularity predicting next-day mood.

**Sad mood	Anxious mood**

*Predictors	Estimates	CI (95%)	Estimates	CI (95%)*

| Intercept | | 1.43 | -0.37 – 3.25 | 2.42 | | 0.43 – 4.43 | |
| --- | --- | --- | --- | --- | --- | --- | --- |
| Age | | 0.07 | -0.05 – 0.18 | -0.02 | | -0.15 – 0.11 | |
| Gender: Female | | 0.16 | -0.08 – 0.39 | 0.34 | | 0.08 – 0.59 | |
| Day of week: Weekend | | -0.16 | -0.22 – -0.10 | -0.12 | | -0.19 – -0.05 | |
| Wave: Wave 2 | | -0.06 | -0.12 – -0.00 | -0.03 | | -0.10 – 0.03 | |
| Wave: Wave 3 | | -0.08 | -0.14 – -0.02 | -0.01 | | -0.08 – 0.06 | |
| Previous day mood | | 0.28 | 0.25 – 0.31 | 0.33 | | 0.30 – 0.36 | |
| Sleep Regularity (centered version) | | -0.30 | -0.71 – 0.10 | -0.28 | | -0.73 – 0.17 | |
| **Random Effects** σ^2^ | | 1.00 |  | 1.00 | |  | |
| τ00 | | 0.35 _study_id_ | | | 0.18 _study_id_ | | |
| τ11 | | 0.18 _study_id.sric_ | | | 0.47 _study_id.sric_ | | |
| ICC | | 0.25 | | | 0.29 | | |
| N | | 102 _study_id_ | | | 97 _study_id_ | | |
|  | Observations | 4087 | | 4004 | | |  |
|  | Marginal R^2^ / Conditional R^2^ | 0.130 / 0.538 | | 0.184 / 0.588 | | |  |

ICC: Intraclass Correlation Coefficient

Influential observations

Based on our registered criteria of a Pareto shape k value above 0.7, we identified no influential observations across the six within-person models.

## Between-person models (Step 2)

Note that the results of this section were yielded by the registered weighted regression approach.

### Table S2.1. Sleep duration couplings predicting internalizing symptoms.

**Depression (CES-DC)	Anxiety (STAIC)**

*Predictors	Estimates	CI (95%)	Estimates	CI (95%)*

| Intercept | -3.98 | -31.04 – 23.79 | -3.99 | -18.91 – 10.76 |
| --- | --- | --- | --- | --- |
| Baseline symptoms | 0.37 | 0.09 – 0.64 | 4.06 | 0.36 – 7.59 |
| Random intercepts | 10.42 | 2.08 – 18.50 | 0.61 | 0.34 – 0.90 |
| Within-person couplings | -0.26 | -19.59 – 18.78 | -0.25 | -9.86 – 9.82 |

Observations	55	52

R^2^ Bayes	0.280	0.426

*Note:* Depression and anxiety were regressed on within-person couplings with sad and anxious mood, respectively.

### Table S2.2. Sleep midpoint couplings predicting internalizing symptoms.

**Depression (CES-DC)	Anxiety (STAIC)**

*Predictors	Estimates	CI (95%)	Estimates	CI (95%)*

| Intercept | 96.67 | 35.73 – 157.51 | 17.69 | 3.43 – 31.34 |
| --- | --- | --- | --- | --- |
| Baseline symptoms | 0.37 | 0.11 – 0.64 | 4.17 | 0.52 – 7.73 |
| Random intercepts | 9.76 | 1.64 – 17.82 | 0.60 | 0.32 – 0.89 |
| Within-person couplings | 0.16 | -19.53 – 20.25 | -0.48 | -10.24 – 9.44 |

Observations	55	52

R^2^ Bayes	0.272	0.429

*Note:* Depression and anxiety were regressed on within-person couplings with sad and anxious mood, respectively.

### Table S2.3. Sleep regularity couplings predicting internalizing symptoms.

**Depression (CES-DC)	Anxiety (STAIC)**

*Predictors	Estimates	CI (95%)	Estimates	CI (95%)*

| Intercept | 16.93 | -2.60 – 36.32 | 6.70 | -16.20 – 29.74 |
| --- | --- | --- | --- | --- |
| Baseline symptoms | 0.34 | 0.07 – 0.61 | 2.93 | -5.33 – 11.10 |
| Random intercepts | 11.65 | 2.23 – 21.13 | 0.59 | 0.32 – 0.87 |
| Within-person couplings | 4.17 | -14.41 – 22.32 | 4.79 | -1.90 – 11.39 |

Observations	55	52

R^2^ Bayes	0.286	0.435

*Note:* Depression and anxiety were regressed on within-person couplings with sad and anxious mood, respectively.

Influential observations

Among the between-person models, one participant (#3) qualified as influential in the prediction of depressive symptoms by couplings of sleep midpoint with sad mood and couplings od sleep regularity and anxious mood. In subsequent sensitivity analyses we removed this participant from the respective models and compared parameters of interest (see Table S10).

## Collinearity between predictors of symptoms

### Table S3. Matrix of zero-order correlations and partial-correlations

|  | **1.** | **2.** | **3.** | **4.** |
| --- | --- | --- | --- | --- |
| **1.** **STAIC (baseline)** | - | .35 | .32 | .55 |
| **2. Random-intercepts** | .14 | - | .98 | .48 |
| **3. Random-slopes (WPCs)** | .06 | .98 | - | .48 |
| **4. STAIC (follow-up)** | .50 | -.01 | .12 | - |

*Note:* Upper triangle coefficients represent zero-order correlations. Lower-triangle coefficients represent first-order partial correlations controlling for random-intercepts. STAIC = State-trait Anxiety Inventory for Children; WPC = Within-Person Coupling.

# Appendix S6. Results based on the registered dataset

## Table S4. Variation in random slopes

| **Model** | **τ2** | **95% CI** | **BF_10_** | **R2c/ R2m** | **WPCR** |
| --- | --- | --- | --- | --- | --- |
| Sad mood (N = 3,591)  Sleep duration | 0.04 | <0.01 to 0.08 | 0.32 | 0.57/0.23 | 0.17 |
| Sleep midpoint | 0.07 | 0.01 to 0.12 | 0.73 | 0.57/0.24 | 0.34 |
| Sleep regularity | 0.32 | 0.02 to 0.68 | 3.93 | 0.57/0.17 | 0.04 |
| Anxious mood (N = 3,534)  Sleep duration | 0.04 | 0.00 to 0.09 | 0.23 | 0.63/0.27 | 0.11 |
| Sleep midpoint | 0.03 | 0.00 to 0.08 | 0.09 | 0.63/0.25 | 0.06 |
| Sleep regularity | 0.48 | 0.06 to 0.85 | 6.71 | 0.63/0.23 | 0.07 |

## Table S5. Between-person associations of sleep-mood couplings and internalizing symptoms

| **Model** | **B** | **95% CI** | **BF10** | **R2** |
| --- | --- | --- | --- | --- |
| Depression (N = 55)  Sad mood - Sleep duration | 1.83 | −17.20 to 21.02 | 1.04 | 0.32 |
| Sad mood - Sleep midpoint | −4.59 | −21.28 to 12.17 | 0.98 | 0.33 |
| Sad mood - Sleep regularity | −0.35 | −15.37 to 14.44 | 0.75 | 0.33 |
| Anxiety (N = 53)  Anxious mood - Sleep duration | −0.18 | −9.92 to 9.60 | 0.99 | 0.53 |
| Anxious mood - Sleep midpoint | −0.19 | −9.80 to 9.67 | 1.02 | 0.52 |
| Anxious mood - Sleep regularity | 3.24 | −2.82 to 9.12 | 1.09 | 0.54 |

# Appendix S7. Sensitivity Analyses

## Figure S6. Prior sensitivity analysis for variation in random slopes

*Note:* Bayes Factors are plotted on a logarithmic y-scale.

## Figure S7. Prior sensitivity analysis for prediction of internalizing symptoms

## Figure S8. Influential observations on the daily level

## Figure S9. Influential observations on the person-level

*Note:* Influential observations are indicated by a Pareto-shape K value above 0.7.

## Table S6. Between-person models without influential observations

| **Model** | **B** | **95% CI** |  | **BF10** | **R2** |
| --- | --- | --- | --- | --- | --- |
| Depression (N = 55)  Sad mood – Sleep midpoint | −5.82 | −23.05 to 11.83 |  | 1.11 | 0.09 |
| Sad mood - Sleep regularity | 3.32 | −11.21 to 17.84 |  | 0.83 | 0.10 |

## Restrictive inclusion

Including only daily data from participants who provided symptom assessments for the Step2 models resulted a reduction of 449 (12.5%) day-night-day sequences involving sad mood and a reduction of 509 (14.4%) data points involving anxious mood. This reduced dataset yielded extreme evidence for the presence of individual differences in couplings of the sleep midpoint and sad mood and strong evidence for couplings involving sleep regularity. The point estimates for the variation of sleep midpoint and sad mood increased by 0.01 points, the variation in couplings between sleep regularity and sad mood increased by 0.12, but the variation in couplings between sleep regularity and anxious mood decreased by 0.1

Table S5.1. Variation in random slopes under restrictive inclusion

| **Model** | **τ^2^** | **95% CI** | | | **BF_10_** | **R2c/ R2m** | | **WPCR** |
| --- | --- | --- | --- | --- | --- | --- | --- | --- |
| Sad mood (N = 3,142)  Sleep duration | 0.05 | 0.01 to 0.09 | | | 2.12 | 0.55/0.21 | | 0.28 |
| Sleep midpoint | 0.08 | 0.04 to 0.11 | | | 1,068. 85 | 0.55/0.22 | | 0.37 |
| Sleep regularity | 0.47 | 0.06 to 1.11 | | | 20.27 | 0.54/0.17 | | 0.06 |
| Anxious mood (N = 3,025)  Sleep duration | 0.03 | <0.01 to 0.06 | | | 0.44 | 0.61/0.25 | | 0.05 |
| Sleep midpoint | 0.03 | <0.00 to 0.07 | | | 0.14 | 0.61/0.25 | | 0.05 |
| Sleep regularity | 0.40 | 0.05 to 0.78 | | | 12.21 | 0.61/0.23 | | 0.04 |
| Table S5.2. Associations of slee | p-mood c | ouplings and inte | | | rnalizing sy | mptoms | |  |
| **Model** | | | **B** | **95% CI** | | | **BF10** | **R2** |
| Depression (N = 55)  Sad mood - Sleep duration | | | −3.07 | −21.73 to 15.53 | | | 1.01 | 0.08 |
| Sad mood - Sleep midpoint | | | −4.76 | −21.92 to 12.48 | | | 1.00 | 0.08 |
| Sad mood - Sleep regularity | | | 2.33 | −7.51 to 11.81 | | | 0.55 | 0.10 |
| Anxiety (N = 53)  Anxious mood - Sleep duration | | | −0.03 | −9.88 to 9.83 | | | 1.00 | 0.37 |
| Anxious mood - Sleep midpoint | | | −0.10 | −10.05 to 9.90 | | | 1.03 | 0.37 |
| Anxious mood - Sleep regularity | | | 2.35 | −4.95 to 9.67 | | | 0.90 | 0.37 |

## Explicit person-mean-centering of the Sleep Regularity Index

### Table S7. Results of person-mean-centered sleep regularity.

| **Model** | **τ2** | **95% CI** | **BF_10_** | **R2c/ R2m** | **WPCR** |
| --- | --- | --- | --- | --- | --- |
| Step-1 models predicting:  Sad mood | 1.03 | 0.20 to 1.74 | 23.47 | 0.55/0.17 | 0.29 |
| Anxious mood | 0.32 | 0.02 to 0.84 | 2.16 | 0.61/0.23 | 0.02 |
| Step-2 models predicting :  Depression | 1.21 | −1.60 to 4.03 | 0.21 | 0.11 | 1.21 |
| Anxiety | −0.15 | −9.53 to 9.40 | 0.93 | 0.38 | −0.15 |

*Note:* PMC = Person-mean-centered

## Alternative symptom outcomes

### Table S8. Average weekly symptoms of depression and anxiety

| **Model** | **B** | **95% CI** | **BF_10_** | **R2** |
| --- | --- | --- | --- | --- |
| Depression (N = 55)  Sad mood - Sleep duration | −0.56 | −16.49 to 15.21 | 1.09 | 0.12 |
| Sad mood - Sleep midpoint | −7.43 | −23.09 to 7.72 | 0.27 | 0.13 |
| Sad mood - Sleep regularity | 5.35 | −1.43 to 12.04 | 0.11 | 0.17 |
| Anxiety (N = 53)  Anxious mood - Sleep duration | −0.29 | −8.32 to 7.74 | 1.08 | 0.30 |
| Anxious mood - Sleep midpoint | −0.57 | −8.76 to 7.50 | 0.84 | 0.31 |
| Anxious mood - Sleep regularity | 4.09 | −0.28 to 8.58 | 0.07 | 0.32 |

### Table S9. Internalizing subscale of the Youth Self-Report

| **Model** | **B** | **95% CI** | **BF_10_** | **R2** |
| --- | --- | --- | --- | --- |
| Sad mood - Internalizing (N = 55)  Sad mood - Sleep duration | −0.21 | −16.86 to 16.40 | 1.03 | 0.70 |
| Sad mood - Sleep midpoint | 0.54 | −15.79 to 16.71 | 1.10 | 0.69 |
| Sad mood - Sleep regularity | −2.55 | −15.29 to 10.12 | 1.70 | 0.70 |
| Anxious mood - Internalizing (N = 53)  Anxious mood - Sleep duration | −0.49 | −16.89 to 16.26 | 1.08 | 0.70 |
| Anxious mood - Sleep midpoint | 0.68 | −16.26 to 17.40 | 1.10 | 0.70 |
| Anxious mood - Sleep regularity | 0.01 | −8.84 to 8.85 | 0.98 | 0.71 |

# Appendix S8. Registered exploratory analyses

## Within-Person Coupling Reliability

Our operationalization of within-person couplings crucially depends on long-term assessments of sleep and daytime mood. To gain insights in the sensitivity of the variation in within-person couplings to the amount of underlying data we re-estimated the parameter of variation across increments of available data starting from the first week of each wave towards the fully protocol as estimated in the main analyses. Estimates of variation in random slopes varied considerably across incremental datasets as well as across waves. However, best average Within-Person Coupling Reliability (Neubauer et al., 2020) scores were obtained by the full dataset (see Tables S7.1-6).

### Table S10.1. Incremental Within-person Coupling Reliability for sleep duration and sad mood

*Note*: Colored cells indicate the selected weeks used for estimation. Upper cell values are point estimates for the variation of random slopes and lower values in parentheses show the Within-Person Coupling Reliability Index. Periods in the same color were aggregated.

### Table S10.2. Incremental Within-person Coupling Reliability for sleep midpoint and sad mood

*Note*: Upper cell values are point estimates for the variation of random slopes and lower values in parentheses show the Within-Person Coupling Reliability Index. Periods in the same color were aggregated.

### Table S10.3. Incremental Within-person Coupling Reliability for sleep regularity and sad mood

*Note*: Upper cell values are point estimates for the variation of random slopes and lower values in parentheses show the Within-Person Coupling Reliability Index. Periods in the same color were aggregated.

### Table S10.4. Incremental Within-person Coupling Reliability for sleep duration and anxious mood

*Note*: Upper cell values are point estimates for the variation of random slopes and lower values in parentheses show the Within-Person Coupling Reliability Index. Periods in the same color were aggregated.

### Table S10.5. Incremental Within-person Coupling Reliability for sleep midpoint and anxious

*Note*: Upper cell values are point estimates for the variation of random slopes and lower values in parentheses show the Within-Person Coupling Reliability Index. Periods in the same color were aggregated.

### Table S10.6. Incremental Within-person Coupling Reliability for sleep midpoint and anxious

*Note*: Upper cell values are point estimates for the variation of random slopes and lower values in parentheses show the Within-Person Coupling Reliability Index. Periods in the same color were aggregated.

## Modeling residual variance and multivariate outcomes

As outlined in our registration, we developed mixed-effects location-scale models (MELSM) and their multivariate extension (M-MELSM; Williams et al., 2020). Both model sets incorporated a random parameter to capture residual variability in daily mood levels. The MMELSM was specifically designed to jointly predict the daily outcomes of sad and anxious moods while also quantifying their covariation. However, neither modeling approach yielded stable results, as evidenced by diverging transitions and an insufficient Effective Sample Size (ESS < 1000).

Additionally, we explored two sets of 3-level models. The first approach, consistent with our registered plan, aimed to estimate random slopes for each wave within participants. The second approach extended this by estimating random slopes for each moment of EMA (Ecological Momentary Assessment) assessment (i.e., morning, noon, afternoon, evening) within participants. Despite these efforts, the Markov Chain Monte Carlo (MCMC) chains failed to converge, as indicated by diverging transitions and low ESS.

## Interactions with within-person couplings

Age, gender, weekend, number of assessment wave were each included as an additional interaction term for the fixed within-person slope. Age and gender were also added to the adjustment set of between-person models. We compared the extended models regarding the registered criteria of Leave-one-out (LOO) Cross-Validation based comparison. Higher Expected Log Pointwise Predictive Density (ELPD) and lower LOO information criterion (LOOIC) indicate better model fit (Vehtari et al., 2017). Only the cross-level interaction of baseline anxiety symptoms (STAIC) with the fixed within person effect of sleep midpoint and regularity yielded clear improvement of model fit, whereas other the other candidate models remained indistinguishable from the model without cross-level interactions (see Tables S8.1-6). However, for the two anxiety-sleep midpoint interactions, Bayes Factors were indecisive (STAIC*midpoint: BF_10_ = 0.39 and STAIC*regularity BF_10_ = 1.22). We did not explore any higherlevel interactions.

### Table S11.1. Model comparison for couplings of sleep duration and sad mood

| Model | ELPD (SE) | LOOIC (SE) |
| --- | --- | --- |
| Main analysis | -3413.1 (54.2) | 6826.2 (108.4) |
| Age | -3413.2 (54.2) | 6826.4 (108.4) |
| Gender | -3413.5 (54.1) | 6827 (108.3) |
| Weekend | -3412.6 (54.2) | 6825.2 (108.3) |
| Block | -3415.1 (54.2) | 6830.2 (108.4) |
| Baseline symptoms | -3412.7 (54.2) | 6825.5 (108.4) |

*Note:* ELPD = Expected Log Pointwise Predictive Density, LOO = Leave-one-out Cross-Validation information criterion (LOOIC)

### Table S11.2. Model comparison for couplings of sleep midpoint and sad mood

| Model | ELPD (SE) | LOOIC (SE) |
| --- | --- | --- |
| Main analysis | -3408.1 (55.1) | 6816.2 (110.1) |
| Age | -3408.5 (55.1) | 6817 (110.2) |
| Gender | -3408.6 (55.1) | 6817.2 (110.1) |
| Weekend | -3408.3 (54.9) | 6816.7 (109.8) |
| Block | -3408.5 (54.8) | 6817 (109.7) |
| Baseline symptoms | -3406.7 (54.8) | 6813.3 (109.6) |

*Note:* ELPD = Expected Log Pointwise Predictive Density, LOO = Leave-one-out Cross-Validation information criterion (LOOIC)

### Table S11.3. Model comparison for couplings of sleep regularity and sad mood

| Model | ELPD (SE) | LOOIC (SE) |
| --- | --- | --- |
| Main analysis | -3414.5 (54.1) | 6829.1 (108.2) |
| Age | -3414.4 (54.2) | 6828.7 (108.3) |
| Gender | -3414.6 (54.1) | 6829.2 (108.2) |
| Weekend | -3414.1 (54.1) | 6828.1 (108.3) |
| Block | -3413.6 (54) | 6827.2 (108.1) |
| Baseline symptoms | -3410.8 (54.1) | 6821.6 (108.2) |

*Note:* ELPD = Expected Log Pointwise Predictive Density, LOO = Leave-one-out Cross-Validation information criterion (LOOIC)

### Table S11.4. Model comparison for couplings of sleep duration and anxious mood

| Model | ELPD (SE) | LOOIC (SE) |
| --- | --- | --- |
| Main analysis | -3687.9 (44.9) | 7375.7 (89.9) |
| Age | -3688.8 (44.9) | 7377.6 (89.9) |
| Gender | -3689.3 (45) | 7378.7 (89.9) |
| Weekend | -3688.9 (45) | 7377.8 (90) |
| Block | -3688.9 (44.9) | 7377.8 (89.8) |
| Baseline symptoms | -3688.6 (44.9) | 7377.1 (89.9) |

*Note:* ELPD = Expected Log Pointwise Predictive Density, LOO = Leave-one-out Cross-Validation information criterion (LOOIC)

### Table S11.5. Model comparison for couplings of sleep midpoint and anxious mood

| Model | ELPD (SE) | LOOIC (SE) |
| --- | --- | --- |
| Main analysis | -4347.2 (50) | 8694.4 (100.1) |
| Age | -4346.5 (50) | 8693.1 (99.9) |
| Gender | -4348 (50.1) | 8695.9 (100.1) |
| Weekend | -4347.6 (50.1) | 8695.3 (100.1) |
| Block | -4346.9 (50.1) | 8693.9 (100.2) |
| **Baseline symptoms** | **-4014.8 (46.9)** | **8029.6 (93.8)** |

*Note:* ELPD = Expected Log Pointwise Predictive Density, LOO = Leave-one-out Cross-Validation information criterion (LOOIC)

### Table S11.6. Model comparison for couplings of sleep regularity and anxious mood

| Model | ELPD (SE) | LOOIC (SE) |
| --- | --- | --- |
| Main analysis | -4345.4 (50.1) | 8690.9 (100.2) |
| Age | -4345.8 (50) | 8691.6 (100.1) |
| Gender | -4346.5 (50.1) | 8692.9 (100.2) |
| Weekend | -4345.9 (50.1) | 8691.8 (100.2) |
| Block | -4343.4 (50.1) | 8686.9 (100.2) |
| **Baseline symptoms** | **-4012.9 (46.9)** | **8025.9 (93.9)** |

*Note:* ELPD = Expected Log Pointwise Predictive Density, LOO = Leave-one-out Cross-Validation information criterion (LOOIC)

## Nonlinear couplings of sleep patterns and daytime mood

Research on the sleep-mood relation has largely investigated linear relations (Hickman et al., 2024). However, there is no convincing reason that linearity holds in every context, for instance, that gains and losses in sleep duration or shifts towards earlier vs. later bedtimes predict daytime mood in the same amount in opposite directions. Nonlinear relations may account for curvilinear effects, that allow expressing the optimal amount of sleep regarding next day mood, as well as asymmetric relations (e.g. exponential, logarithmic) that express accelerating response in daytime mood only in one direction (e.g. sleep loss) but not the other (e.g., sleep gains). Nonlinear relations were previously addressed in studies of sleep mood relation in adults (e.g.; Lee et al., 2024; Sayre et al., 2021) as well as in other fields of withinperson field research, such as stress reactivity (Rush et al., 2024). In our exploratory analyses, we tested potential quadratic, exponential, and logarithmic relations by transforming the sleep-pattern predictors accordingly. For couplings involving sleep duration and sleep midpoint with both sad and anxious mood, models showed improved model fit. However, fixed and random within-person effects of these models were estimated narrowly to zero (all 95% Credible Intervalls [-0.1; 0.1]).

### Table S12.1. Model comparison for couplings of sleep duration and sad mood

| Model | ELPD (SE) | LOOIC (SE) |
| --- | --- | --- |
| Linear | -3960.9 (61.4) | 7921.8 (122.7) |
| Quadratic | -3416.5 (54.1) | 6833 (108.3) |
| **Exponential** | **-1483.8 (32.8)** | **2967.6 (65.7)** |
| Logarithmic | -3416.3 (54.2) | 6832.6 (108.4) |

### Table S12.2. Model comparison for couplings of sleep midpoint and sad mood

| Model | ELPD (SE) | LOOIC (SE) |
| --- | --- | --- |
| Linear | -3956.1 (61.8) | 7912.2 (123.7) |
| Quadratic | -3415.1 (54.2) | 6830.2 (108.3) |
| **Exponential** | **-1377.2 (29.2)** | **2754.3 (58.3)** |
| Logarithmic | -3412.2 (54.3) | 6824.4 (108.6) |

### Table S12.3. Model comparison for couplings of sleep regularity and sad mood

| Model | ELPD (SE) | LOOIC (SE) |
| --- | --- | --- |
| Linear | -3960.7 (61) | 7921.4 (121.9) |
| Quadratic | -3414.2 (54.1) | 6828.4 (108.2) |
| Exponential | -3410.2 (54.3) | 6820.5 (108.6) |
| Logarithmic | -3410.8 (54.2) | 6821.6 (108.4) |

### Table S12.4. Model comparison for couplings of sleep duration and anxious mood

| Model | ELPD (SE) | LOOIC (SE) |
| --- | --- | --- |
| Linear | -4342.2 (50.2) | 8684.5 (100.4) |
| Quadratic | -3692.8 (45.3) | 7385.6 (90.7) |
| **Exponential** | **-1602.3 (29.5)** | **3204.5 (59.1)** |
| Logarithmic | -3690 (45) | 7380.1 (90) |

### Table S12.5. Model comparison for couplings of sleep duration and anxious mood

| Model | ELPD (SE) | LOOIC (SE) |
| --- | --- | --- |
| Linear | -4347.2 (50) | 8694.4 (100.1) |
| Quadratic | -3689.2 (44.9) | 7378.4 (89.8) |
| **Exponential** | **-1484.3 (27.8)** | **2968.7 (55.6)** |
| Logarithmic | -3689.6 (44.9) | 7379.2 (89.9) |

### Table S12.6. Model comparison for couplings of sleep regularity and anxious mood

| Model | ELPD (SE) | LOOIC (SE) |
| --- | --- | --- |
| Linear | -4345.4 (50.1) | 8690.9 (100.2) |
| Quadratic | -3687.6 (44.9) | 7375.2 (89.8) |
| Exponential | -3688.5 (44.9) | 7377.1 (89.9) |
| Logarithmic | -3688.2 (44.9) | 7376.5 (89.8) |

## Pubertal maturation

We also investigated a baseline measure of self-reported pubertal development (PPDS; Petersen et al., 1988) as an alternative indicator for maturation (Kirshenbaum et al., 2023). Pubertal stage did not significantly interact with the fixed within-person effect of sleep metrics on next-day mood (Table S10). Pubertal stage also did not interact with the effect of withinperson couplings on symptoms of depression or anxiety.

### Table S13. Interaction of pubertal development with sleep patterns

| **Model** | **Inter- action** | **95% CI** | **BF_10_** | **τ^2^** | **95% CI** | **BF_10_** | **R2c/ R2m** | **WPCR** |
| --- | --- | --- | --- | --- | --- | --- | --- | --- |
| Sad mood  Sleep duration | < 0.01 | -0.01 to 0.01 | <0.01 | 0.04 | 0.00 to 0.08 | 0.41 | 0.56/0.20 | 0.19 |
| Sleep midpoint | < 0.01 | -0.02 to 0.01 | <0.01 | 0.07 | 0.03 to 0.11 | 25.45 | 0.56/0.19 | 0.27 |
| Sleep regularity^[1]^ | - | - | - | - | - | - | - | - |
| Anxious mood Sleep duration | < 0.01 | -0.01 to 0.01 | <0.01 | 0.05 | 0.01 to 0.09 | 0.94 | 0.62/0.23 | 0.18 |
| Sleep midpoint | < 0.01 | -0.02 to 0.01 | <0.01 | 0.04 | 0.00 to 0.08 | 0.47 | 0.62/0.22 | 0.08 |
| Sleep regularity^1^ | - | - | - | - | - | - | - | - |

### Table S14. Interaction of pubertal development with sleep-mood couplings predicting internalizing symptoms

| **Model** | **B** | **95% CI** | **BF_10_** | **Inter- action** | **95% CI** | **BF_10_** | **R2** |
| --- | --- | --- | --- | --- | --- | --- | --- |
| Depression (N = 55)  Sad mood - Sleep duration | −0.47 | −20.11 to 19.13 | 0.38 | -2.50 | -7.93 to 3.07 | 1.01 | 0.13 |
| Sad mood - Sleep midpoint | 0.27 | −19.00 to 19.69 | 0.36 | -2.10 | -5.05 to 0.87 | 1.01 | 0.15 |
| Sad mood - Sleep regularity | −1.28 | −19.84 to 17.46 | 0.13 | 0.85 | -1.14 to 2.81 | 1.01 | 0.13 |
| Anxiety (N = 53)  Anxious mood - Sleep duration | −0.06 | −10.03 to 9.77 | 0.27 | -0.17 | -5.97 to 5.72 | 1.02 | 0.39 |
| Anxious mood - Sleep midpoint | −0.09 | −9.89 to 9.66 | 0.64 | -4.15 | -15.44 to 6.91 | 0.99 | 0.40 |
| Anxious mood - Sleep regularity | 0.77 | −8.48 to 10.07 | 0.04 | 0.25 | -0.51 to 0.98 | 0.91 | 0.40 |

## Alternative sleep variables

Based in the availability of subjectively reported sleeponset and wakeup times in the first EMA survey of each day, we computed subjective sleep duration and midpoint. We also considered subjectively reported sleep quality as an alternative predictor. Finally, we also quantified daily deviations from chronotype as the difference from each night’s sleep midpoint to the average sleep midpoint on school-free days. The latter is commonly regarded as a proxy for chronotype, though we could not control for any restrictions on school-free days such as training sessions or other appointments (Roenneberg et al., 2019).

### Table S15. Variation in random slopes for alternative sleep indicators

| **Model** | **N** | **τ^2^** | **95% CI** | **BF_10_** | **R2c/ R2m** | **WPCR** |
| --- | --- | --- | --- | --- | --- | --- |
| Sad mood  Self-reported sleep duration | 3,485 | 0.03 | < 0.01 to 0.06 | 0.42 | 0.53/0.21 | 0.10 |
| Self-reported sleep midpoint | 4,170 | 0.06 | 0.02 to 0.10 | 26.22 | 0.55/0.24 | 0.30 |
| Self-reported sleep quality | 4,197 | 0.08 | 0.06 to 0.11 | >1,000 | 0.56/0.31 | 0.49 |
| Deviation from chronotype | 3,528 | 0.05 | 0.01 to 0.10 | 0.91 | 0.53/0.22 | 0.22 |
| Anxious mood  Self-reported sleep duration | 3,485 | 0.03 | <0.01 to 0.07 | 0.25 | 0.58/0.23 | 0.08 |
| Self-reported sleep midpoint | 4,170 | 0.06 | 0.02 to 0.09 | 7.66 | 0.58/0.21 | 0.20 |
| Self-reported sleep quality | 4,197 | 0.06 | 0.02 to 0.09 | 5.53 | 0.58/0.28 | 0.26 |
| Deviation from chronotype | 3,528 | 0.04 | <0.01 to 0.09 | 0.57 | 0.58/0.22 | 0.12 |

### Table S16. Associations of sleep-mood couplings and internalizing symptoms

| **Model** | **B** | **95% CI** | **BF_10_** | **R^2^** |
| --- | --- | --- | --- | --- |
| Depressive symptoms (N = 52)  Self-reported sleep duration | −0.84 | −20.64 to 19.15 | 1.02 | 0.07 |
| Self-reported sleep midpoint | −2.91 | −20.87 to 15.19 | 0.99 | 0.07 |
| Self-reported sleep quality | −3.52 | −19.57 to 12.47 | 0.90 | 0.08 |
| Deviation from chronotype | −3.32 | −22.01 to 15.50 | 1.04 | 0.10 |
| Anxiety symptoms (N = 51) Self-reported sleep duration | −0.36 | −10.03 to 9.36 | 0.98 | 0.38 |
| Self-reported sleep midpoint | −0.89 | −10.50 to 8.91 | 1.03 | 0.36 |
| Self-reported sleep quality | −1.84 | −11.23 to 7.63 | 1.07 | 0.37 |
| Deviation from chronotype | −0.62 | −10.25 to 9.09 | 0.98 | 0.36 |

### Figure S10. Correlations among daily mood and sleep variables

### Figure S11. Partial correlations among daily mood and sleep variables

## Circadian preference

Finally, we tested circadian preference as a cross-level moderator of sleep’s daily couplings with day-time mood. Circadian preference was measured with the Morningness Eveningness Scale for Children (Caci et al., 2005; Carskadon et al., 1993), a 10 item self-report inquiring preferred clock times for physical activity, academic effort, and sleep. Eight items are coded on a 4-point Likert scale and the remaining two on a 5-point Likert scale. The sum score ranges from 10 to 42. We found no fixed within-person effects to be substantially influenced by circadian preference, with sleep regularity couplings not converging at the initial setup (4*4000 iterations). The association of sleep-mood couplings with symptom severity was also unaffected by circadian preference.

### Table S17. Interaction of circadian preference with sleep patterns

| **Model** | **Inter- action** | **95% CI** | **BF_10_** | **τ^2^** | **95% CI** | **BF_10_** | **R2c/ R2m** | **WPCR** |
| --- | --- | --- | --- | --- | --- | --- | --- | --- |
| Sad mood  Sleep duration | <0.01 | -0.01 to 0.01 | <0.01 | 0.04 | <0.01 to 0.08 | 1.23 | 0.55/0.24 | 0.19 |
| Sleep midpoint | <0.01 | -0.01 to 0.01 | <0.01 | 0.07 | 0.02 to 0.10 | 2.31 | 0.55/0.22 | 0.24 |
| Sleep regularity^[2]^ | - | - | - | - | - | - | - | - |
| Anxious mood Sleep duration | <0.00 | <0.01 to 0.01 | <0.01 | 0.04 | <0.00 to 0.08 | 0.77 | 0.62/0.28 | 0.14 |
| Sleep midpoint | 0.01 | <0.01 to 0.01 | <0.01 | 0.03 | <0.00 to 0.08 | 0.51 | 0.62/0.27 | 0.07 |
| Sleep regularity^1^ | - | - | - | - | - | - | - | - |

### Table S18. Interaction of circadian preference with sleep-mood couplings predicting internalizing symptoms

| **Model** | **B** | **95% CI** | **BF_10_** | **Inter- action** | **95% CI** | **BF_10_** | **R2** |
| --- | --- | --- | --- | --- | --- | --- | --- |
| Depression (N = 55)  Sad mood - Sleep duration | 0.03 | −19.58 to 20.03 | 1.02 | 0.13 | -28.95 to 29.94 | 0.97 | 0.17 |
| Sad mood - Sleep midpoint | −0.13 | −19.92 to 20.10 | 0.94 | -0.06 | -16.05 to 15.64 | 0.66 | 0.18 |
| Sad mood - Sleep regularity | 0.01 | −19.73 to 19.87 | 1.01 | -0.01 | -1.17 to 1.20 | 0.04 | 0.17 |
| Anxiety (N = 53)  Anxious mood - Sleep duration | 0.03 | −9.88 to 9.91 | 1.02 | -0.12 | -8.19 to 7.76 | 0.29 | 0.39 |
| Anxious mood - Sleep midpoint | −0.06 | −10.07 to 9.78 | 1.04 | -0.09 | -26.66 to 26.17 | 0.88 | 0.39 |
| Anxious mood - Sleep regularity | 0.02 | −9.56 to 9.81 | 1.02 | 0.01 | -5.07 to 5.01 | 0.17 | 0.37 |

# References

Achenbach, T. M., & Rescorla, L. A. (2001). *Manual for the ASEBA School-Age Forms and*

*Profiles*. University of Vermont Research Center for Children, Youth, & Families.

Caci, H., Robert, P., Dossios, C., & Boyer, P. (2005). L’échelle de matinalité pour enfants et adolescents: Propriétés psychométriques et effet du mois de naissance. *L’Encéphale*, *31*(1), 56–64. https://doi.org/10.1016/S0013-7006(05)82372-3

Carskadon, M. A., Vieira, C., & Acebo, C. (1993). Association between Puberty and Delayed Phase Preference. *Sleep*, *16*(3), 258–262. https://doi.org/10.1093/sleep/16.3.258

Dzubur, E., Ponnada, A., Nordgren, R., Yang, C.-H., Intille, S., Dunton, G., & Hedeker, D. (2020). MixWILD: A program for examining the effects of variance and slope of time-varying variables in intensive longitudinal data. *Behavior Research Methods*, *52*(4), 1403–1427. https://doi.org/10/gnx4gb

Hickman, R., D’Oliveira, T. C., Davies, A., & Shergill, S. (2024). Monitoring Daily Sleep, Mood, and Affect Using Digital Technologies and Wearables: A Systematic Review. *Sensors*,

*24*(14), Article 14. https://doi.org/10.3390/s24144701

Kirshenbaum, J. S., Coury, S. M., Colich, N. L., Manber, R., & Gotlib, I. H. (2023). Objective and subjective sleep health in adolescence: Associations with puberty and affect. *Journal of Sleep Research*, *32*(3), e13805. https://doi.org/10.1111/jsr.13805

Lee, S. A., Mukherjee, D., Rush, J., Lee, S., & Almeida, D. M. (2024). Too little or too much: Nonlinear relationship between sleep duration and daily affective well-being in depressed adults. *BMC Psychiatry*, *24*(1), 323. https://doi.org/10.1186/s12888-02405747-7

Neubauer, A. B., Voelkle, M. C., Voss, A., & Mertens, U. K. (2020). Estimating Reliability of

Within-Person Couplings in a Multilevel Framework. *Journal of Personality Assessment*,

*102*(1), 10–21. https://doi.org/10.1080/00223891.2018.1521418

Parker, R. M. A., Leckie, G., Goldstein, H., Howe, L. D., Heron, J., Hughes, A. D., Phillippo, D.

M., & Tilling, K. (2021). Joint Modeling of Individual Trajectories, Within-Individual

Variability, and a Later Outcome: Systolic Blood Pressure Through Childhood and Left

Ventricular Mass in Early Adulthood. *American Journal of Epidemiology*, *190*(4), 652–

662. https://doi.org/10.1093/aje/kwaa224

Petersen, A. C., Crockett, L., Richards, M., & Boxer, A. (1988). A self-report measure of pubertal status: Reliability, validity, and initial norms. *Journal of Youth and Adolescence*, *17*(2), 117–133. https://doi.org/10/cwxw7p

Rieck, T., Jackson, A., Martin, S. B., Petrie, T., & Greenleaf, C. (2013). Health-Related Fitness,

Body Mass Index, and Risk of Depression among Adolescents. *Medicine & Science in Sports & Exercise*, *45*(6), 1083. https://doi.org/10.1249/MSS.0b013e3182831db1

Roenneberg, T., Pilz, L. K., Zerbini, G., & Winnebeck, E. C. (2019). Chronotype and Social Jetlag:

A	(Self-)	Critical	Review.	*Biology*,	*8*(3),	Article	3.

https://doi.org/10.3390/biology8030054

Rush, J., Ong, A. D., Piazza, J. R., Charles, S. T., & Almeida, D. M. (2024). Too little, too much, and “just right”: Exploring the “goldilocks zone” of daily stress reactivity. *Emotion*,

*24*(5), 1249–1258. https://doi.org/10.1037/emo0001333

Sayre, G. M., Grandey, A. A., & Almeida, D. M. (2021). Does sleep help or harm managers’ perceived productivity? Trade-offs between affect and time as resources. *Journal of*

*Occupational	Health	Psychology*,	*26*(2),	127–141.

https://doi.org/10.1037/ocp0000192

Shen, L., Wiley, J. F., & Bei, B. (2022). Sleep and affect in adolescents: Bidirectional daily associations over 28-day ecological momentary assessment. *Journal of Sleep Research*,

*31*(2), e13491. https://doi.org/10.1111/jsr.13491

Turgeon, L., & Chartrand, É. (2003). Psychometric Properties Of The French Canadian Version

Of The State-Trait Anxiety Inventory For Children. *Educational and Psychological*

*Measurement*, *63*(1), 174–185. https://doi.org/10.1177/0013164402239324

Vehtari, A., Gelman, A., & Gabry, J. (2017). Practical Bayesian model evaluation using leaveone-out cross-validation and WAIC. *Statistics and Computing*, *27*(5), 1413–1432. https://doi.org/10/gdj2kz

Williams, D. R., Martin, S. R., Liu, S., & Rast, P. (2020). Bayesian Multivariate Mixed-Effects

Location Scale Modeling of Longitudinal Relations Among Affective Traits, States, and Physical Activity. *European Journal of Psychological Assessment*, *36*(6), 981–997.

https://doi.org/10/gh432c

1. models did not converge
2. models did not converge
